# *Chlorella vulgaris* genome assembly and annotation reveals the molecular basis for metabolic acclimation to high light conditions

**DOI:** 10.1111/tpj.14508

**Published:** 2019-09-24

**Authors:** Michela Cecchin, Luca Marcolungo, Marzia Rossato, Laura Girolomoni, Emanuela Cosentino, Stephan Cuine, Yonghua Li‐Beisson, Massimo Delledonne, Matteo Ballottari

**Affiliations:** ^1^ Dipartimento di Biotecnologie Università di Verona Strada Le Grazie 15 37134 Verona, Italy; ^2^ Institute of Biosciences and Biotechnologies of Aix‐Marseille, UMR7265 Aix‐Marseille University CEA CNRS CEA Cadarache Saint‐Paul‐lez Durance F‐13108 France

**Keywords:** biofuels, carotenoids, *Chlorella vulgaris*, horizontal gene transfer, lipids, microalgae, motility, fatty acid synthase

## Abstract

*Chlorella vulgaris* is a fast‐growing fresh‐water microalga cultivated on the industrial scale for applications ranging from food to biofuel production. To advance our understanding of its biology and to establish genetics tools for biotechnological manipulation, we sequenced the nuclear and organelle genomes of *Chlorella vulgaris* 211/11P by combining next generation sequencing and optical mapping of isolated DNA molecules. This hybrid approach allowed us to assemble the nuclear genome in 14 pseudo‐molecules with an N50 of 2.8 Mb and 98.9% of scaffolded genome. The integration of RNA‐seq data obtained at two different irradiances of growth (high light, HL versus low light, LL) enabled us to identify 10 724 nuclear genes, coding for 11 082 transcripts. Moreover, 121 and 48 genes, respectively, were found in the chloroplast and mitochondrial genome. Functional annotation and expression analysis of nuclear, chloroplast and mitochondrial genome sequences revealed particular features of *Chlorella vulgaris*. Evidence of horizontal gene transfers from chloroplast to mitochondrial genome was observed. Furthermore, comparative transcriptomic analyses of LL versus HL provided insights into the molecular basis for metabolic rearrangement under HL versus LL conditions leading to enhanced *de novo* fatty acid biosynthesis and triacylglycerol accumulation. The occurrence of a cytosolic fatty acid biosynthetic pathway could be predicted and its upregulation upon HL exposure was observed, consistent with the increased lipid amount under HL conditions. These data provide a rich genetic resource for future genome editing studies, and potential targets for biotechnological manipulation of *Chlorella vulgaris* or other microalgae species to improve biomass and lipid productivity.

## Significance Statement

Microalgae cultivation is one of the most promising strategies for novel food or biofuel production. High‐quality genome information is required for understanding algae biology, for their biotechnological optimization and for genome editing applications. Here, the genome of *Chlorella vulgaris*, one of the green algae species with the highest productivity potential, is presented and functionally annotated, revealing notable features among *Chlorophyta*.

## Introduction

Photosynthetic conversion of light into chemical energy for CO_2_ fixation is the primary process for biomass production on our planet. The improvement of photosynthetic biomass production is thus critical to satisfy the world demand for food and energy (Ort *et al*., 2015), which requires fundamentally improving photosynthetic efficiency (Ort *et al*., 2015; Berteotti *et al*., 2016; Kromdijk *et al*., 2016; Kirst *et al*., 2017). Among the organisms with the highest photosynthetic efficiency observed in real cultivation cases, microalgae scored efficiencies of 1–3%, although this is still significantly lower compared with their maximum potential (9–11%) (Walker, 2009), highlighting the potential for further improvement. In addition, unicellular microalgae are promising platforms for biomass, food or biofuel production: they can be cultivated in none‐arable land in open ponds or in closed photobioreactors potentially employing waste products and wastewater‐derived effluents as nutrients (Lum *et al*., 2013). However, biotechnological manipulation of microalgae to further boost biomass and metabolite productivity require the availability of high‐quality genomes and transcriptomes (Merchant *et al*., 2007; Radakovits *et al*., 2012; Vieler *et al*., 2012; Ajjawi *et al*., 2017; Roth *et al*., 2017). High‐quality genomes are especially critical considering the newly developed technology of genome editing methods (Naduthodi *et al*., 2018).

Among the many candidates of algal strains for biotechnological applications, a genus of considerable interest is *Chlorella* (Blanc *et al*., 2010; Juneja *et al*., 2016; Zuniga *et al*., 2016; Sarayloo *et al*., 2017; Arriola *et al*., 2018). Several species of *Chlorella* have been proposed or have already been used commercially over the past 40 years as a food and feed supplement because of their fast growth and their high resistance to biotic and abiotic stresses (Lum *et al*., 2013). *Chlorella vulgaris* is one of the most cultivated species at the industrial scale because of the high biomass yield and the possibility to grow either in autotrophic or mixotrophic conditions, in the latter case with the addition of reduced carbon source to further improve the biomass yield (Lv *et al*., 2010; Zuniga *et al*., 2016).

Lipid metabolism is among the most investigated topics for microalgae industrial application with the aim to produce biofuels or biomass with high nutrition content. *De novo* fatty acid biosynthesis occurs in plant cells mainly in the chloroplast catalyzed by fatty acid synthase type II (FAS2) multisubunit complex, while animals and fungi possess FAS type I complexes (FAS1) located in the cytosol which appear as large multi‐enzyme complexes composed by one or two large polypeptide chains (Alboresi *et al*., 2016). In type I system, the constituent catalytic components are covalently linked in multifunctional megasynthases, whereas in type II system, the catalytic components are independent monofunctional polypeptides (Smith and Tsai, 2007). The occurrence of FAS1‐like complexes in algal cell have already been suggested in the oleaginous species *Nannochloropsis oceanica* and *Nannochloropsis gaditana* (Vieler *et al*., 2012; Alboresi *et al*., 2016), but not yet in the green lineage, where, however, the genome resources available for green algae are limited. For *Chlorella* genus, only a few species for which high‐quality or draft genomes are available (Blanc *et al*., 2010, 2012; Gao *et al*., 2014; Roth *et al*., 2017; Arriola *et al*., 2018; Guarnieri *et al*., 2018; Hovde *et al*., 2018). In the specific case of *C. vulgaris*, a fragmented genome of 113 scaffolds has been recently reported (Guarnieri *et al*., 2018), and the high‐number of scaffolds jeopardizes an effective implementation of genome editing methods. Indeed, the reported *C. vulgaris* genome has been obtained only based on short‐reads produced with Illumina (San Diego, CA, USA) sequencing, whose assembly is challenging and error‐prone (Yoshinaga *et al*., 2018). Moreover several questions remained unsolved, such as the presence of genes involved in sexual reproduction (Merchant *et al*., 2007; Blanc *et al*., 2010; Roth *et al*., 2017) or the molecular basis for fatty acid biosynthesis (Vieler *et al*., 2012; Alboresi *et al*., 2016). In this work, in order to unravel the genetic information underlying *C. vulgaris* features, a combination of different sequencing technologies and optical mapping led to the reconstruction at near‐chromosome level of the nuclear, chloroplast and mitochondrial genomes of *C. vulgaris 211/11P* strain. Moreover, we provide functional annotation of the genomes with the help of comparative RNA‐seq analyses of strains grown under two most encountered conditions, that is, low light (LL) versus high light (HL).

## Results

### *Chlorella vulgaris* biomass productivity and composition

**Figure 1:**
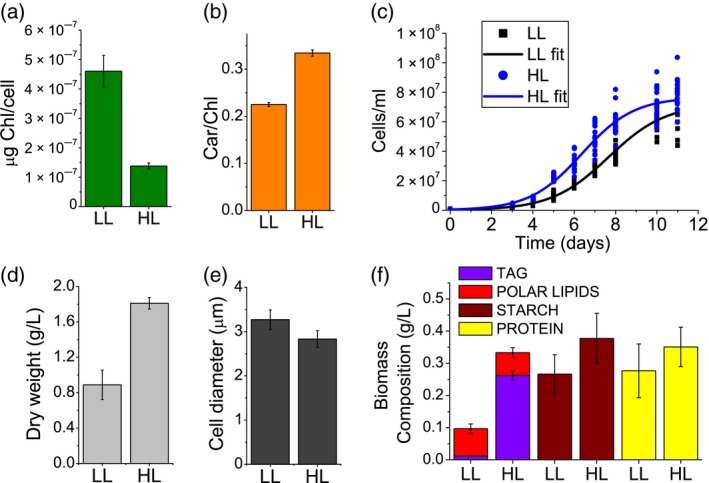
Growth curves, productivity and biomass composition of *Chlorella vulgaris* 211/11P in low light (LL) compared with high light (HL). (a, b) Chlorophyll (Chl) content per cell (a) and carotenoid to chlorophyll ratio (b) in LL and HL. (c) Growth curves of cells grown in LL and HL fitted by sigmoidal function (Hill function). (d) Dry weight of total biomass harvested at the end of the growth curves reported in (a). (e) Average cell diameter at the end of the growth curves reported in (c). (f) Biomass composition analysis in terms of lipids, proteins and starch. Lipid content are indicated either as triacylglycerol (TAG) or as polar lipids (PL, the sum of major membrane lipids). Error bars are reported in terms of standard deviation (*n* = 3 for data reported in a, b, e, f; *n* = 10 for data reported in (c) and (d).

**Figure 2:**
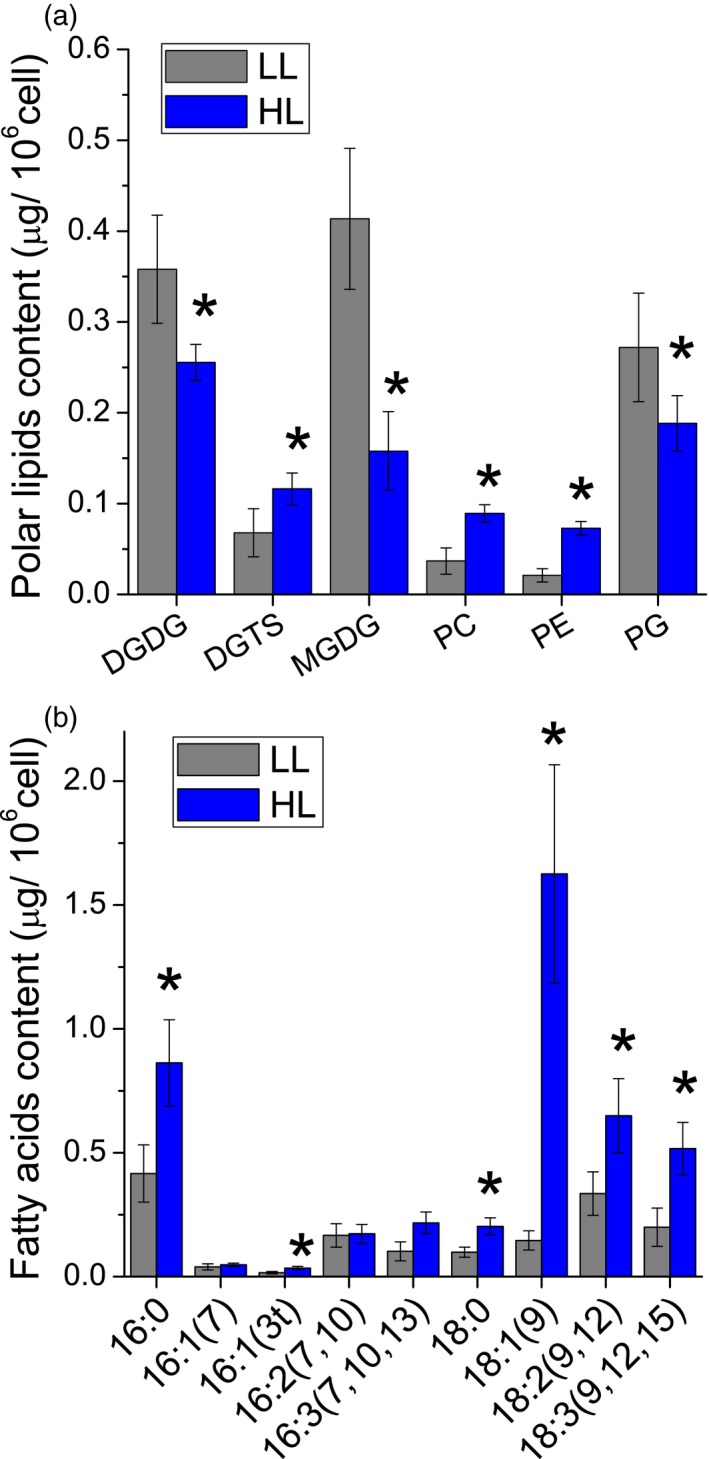
Polar lipid and fatty acid composition in *Chlorella vulgaris* 211/11P cells grown in LL versus HL conditions. (a) A given amount of lipid extracts, harvested from LL‐ or HL‐grown conditions, was subjected to thin layer chromatography to investigate polar lipid profile. (b) Fatty acid profiles obtained by gas chromatography techniques as detailed in Materials and methods. Data are means of three biological replicates with standard deviation shown. Significantly different values in HL versus LL are indicated by * (*P* < 0.05). MGDG, monogalactosyldiacylglycerol; DGDG, digalactosyldiacylglycerol; PG, phosphatidylglycerol; PE, phosphatidylethanolamine; PC, phosphatidylcholine; DGTS, diacylglycerol *N*,*N*,*N*‐trimethylhomoserine.

*Chlorella vulgaris* 211/11P was grown photoautotrophically under LL (70 μmol m^2^ sec^−1^) or HL (1000 μmol m^2^ sec^−1^, HL), to evaluate its biomass productivity and composition. Cultivation in HL conditions caused a strong decrease in chlorophyll and an increase in carotenoids/chlorophyll ratio (Figure 1a). This is consistent with previous report for other green algae species as a mechanism to improve photoprotection and decrease the risk of photodamage (Bonente *et al*., 2012). Growth in HL was faster compared with the LL conditions (Figure 1b). Accordingly, the dry weight harvested when cell density reached the stationary phase was higher for cells grown in HL compared with cells grown in LL (Figure 1c). Cell diameter nevertheless remained similar in LL versus HL conditions (Figure 1c): the two‐fold increase in biomass accumulation observed in HL was thus related to a combined effect of increased cell density and, more relevant, of increased weight of individual cell, likely due to different biomass composition. As reported in Figure 1(d), starch and protein content per cell were not significantly different in LL compared with HL. Strikingly, HL‐grown cells showed a strong increase in lipid accumulation. In particular, the triacylglycerol (TAG) fraction of the total lipid in the cell was increased from 12% in LL to 79% in HL. The increase in TAG content was accompanied by strong decrease in monogalactosyldiacylglycerol (MGDG), digalactosyldiacylglycerol (DGDG) and the phospholipid phosphatidylglycerol (PG) (Figure 2a). This result is consistent with the reduced chlorophyll content per cell observed in HL, being MGDG, DGDG, sulfoquinovosyldiacylglycerol (SQDG, not detectable in *C. vulgaris* 211/11P) and PG the main lipid present in the thylakoid membranes where chlorophyll binding proteins are embedded (Kobayashi, 2016). The fatty acid profile of cells grown in LL versus HL condition is reported in Figure 2(b): HL‐grown cells present a strong increase in palmitic acid (16:0), oleic acid (18:1), linoleic acid (18:2) and α‐linolenic acid (18:3) with oleic acid as the most abundant fatty acid in HL‐grown cells (Figure 2b).

### Development of a high‐quality reference genome sequence of *Chlorella vulgaris*

**Figure 3:**
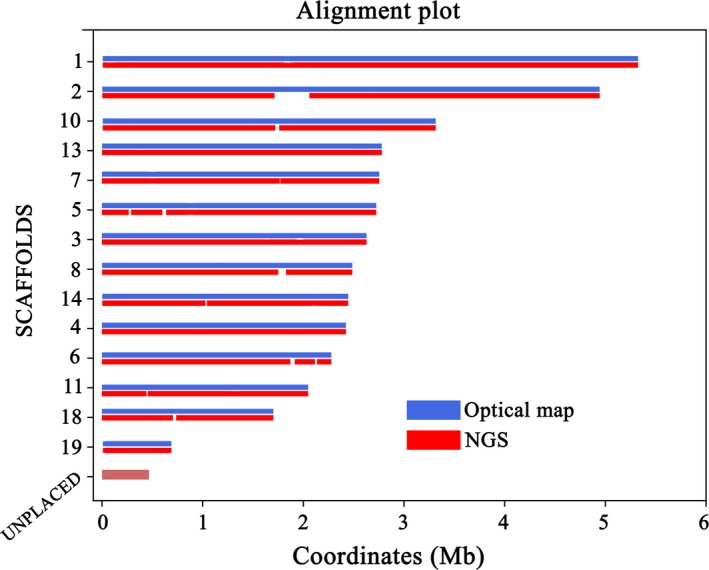
Assembled *Chlorella vulgaris* 211/11P nuclear genome. *Chlorella vulgaris* 211/11P genome was assembled in 14 pseudo‐molecules based on integration of next generation sequencing (NGS) and optical maps as described in the main text. The resulting gaps in the assembled genome are reported as white spaces in the NGS data. Unplaced contigs are reported representing <1.1% of the *Chlorella vulgaris* 211/11P genome.

In order to investigate the genetic basis underlying the highly productive phenotype of *C. vulgaris* 211/11P, we have sequenced, assembled and functionally annotated its nuclear genome. Genome assembly was obtained by integrating different genomic approaches displaying complementary features, i.e., PacBio producing long‐reads, Illumina for accurate short‐reads and Bionano optical mapping providing high scaffolding power. Genome sequencing analysis was conducted initially predicting a potential genome size of ~50 Mb, as for other *Chlorella* spp. (Blanc *et al*., 2010; Arriola *et al*., 2018; Guarnieri *et al*., 2018). High coverage (~128×) raw PacBio reads (Table S1) were assembled into a draft genome assembly of 39.8 Mb (Table S2), consisting of 641Kb and N50 of 1.8 Mb. To improve the quality of the assembled genome, Illumina paired‐end reads (~50×, Table S1), as well as raw PacBio reads, were aligned to the PacBio‐based assembly to correct sequencing errors: 3076 single‐nucleotide variants (SNVs) and 32 821 small insertions and deletions (InDel) were corrected, whereas the remaining 81 SNV and 190 InDel account only for the 0.0007% of the reconstructed genome (Table S3). The resulting polished PacBio‐based contigs were anchored into a nearly chromosome‐scale assembly by integrating optical mapping data (~1400×) obtained using the Bionano Genomics (San Diego, CA, USA) technology (Figure S1 and Table S1). As reported in Table S1, the integration of Bionano data resulted into a genome assembly where 26 of the contigs obtained from PacBio data were anchored into 14 scaffolds (Figure 3) with an N50 value of 2.8 Mb and the longest scaffold of 5.4 Mb. Eight unplaced contigs were identified by subsequent manual analysis as part of the chloroplast and the mitochondrial genomes, and they were therefore removed from the nuclear genome assembly, remaining 29 unplaced contigs that counted only for <1.1%. The 14 scaffolds of the nuclear genome contained 98.9% of the assembled *C. vulgaris* 211/11P genome, i.e. the highest percentage when compared with other algal genomes available (Table 1). The generated assembly represents a greater than 100‐fold improvement in contiguity compared with the previously published assembly of *C. vulgaris* UTEX395 (Table S4) and it has the highest N50 among other algal genomes of similar size as *Chromochloris zofingiensis* (Roth *et al*., 2017) and *Chlorella variabilis* (Blanc *et al*., 2010) (Table 1).

**Table 1:** Comparison of *Chlorella vulgaris* 211/11P genome with other known microalgae genomes

	*Chlorella vulgaris 211/11P* (this work)	*Chromochloris zofingiensis* (Roth *et al*., 2017)	*Chlamydomonas reinhardtii* (v5.5) (Merchant *et al*., 2007; Blaby *et al*., 2014)	*Chlorella variabilis* NC64A (Blanc *et al*., 2010)	*Nannochloropsis gaditana* B‐31 (Corteggiani Carpinelli *et al*., 2014)	*Chlorella sorokiniana 1230* (Hovde *et al*., 2018)
Sequenced genome size	40 Mbp	57 Mbp	107 Mbp	46.2 Mbp	26.3 Mbp	58.5 Mbp
Genome technologies	PacBio + BioNano + Illumina	PacBio + OpGen + Illumina	Sanger + 454 + BAC + genetic map	Sanger WGS	454 + SOLiD + BAC	PacBio + Illumina
No of scaffold	14	19	17 chromosomes	30	21	20
% scaffolded genome	98.9%	95.4%	98.2%	89%	92.2%	100%
Scaffold N50	2.8 Mbp	3.72 Mbp	7 Mbp	1.5 Mbp	1 Mbp	3.82 Mbp
% G+C	61%	51%	64%	67%	54.2%	63.8%
No of genes	10 724	15 274	17 741	9791	10 646	12 871
Exon average length (bp)	194	291	261	170	449	152
Intron average Length (bp)	207	267	269	209	178	215
Ave Exons Per transcript	8.12	5	8.5	7.3	2.71	10.9

### *Chlorella vulgaris* nuclear genome annotation and phylogenetic analysis

Identification of genes present in the assembled *C. vulgaris* 211/11P genome was performed by integration of directional RNA‐seq data obtained from *C. vulgaris* 211/11P cells cultivated in LL and HL into the gene annotation pipeline. Genome annotation identified 10 724 genes, coding for 11 082 transcripts with an average length of 3062 bp and 8.12 exons per gene on average (Table 1). The gene models predicted for *C. vulgaris* 211/11P were used to determine codon usage (Table S5), which is found similar to the codon usage of *C. reinhardtii* (Merchant *et al*., 2007). The number of protein‐coding genes is significantly higher compared with the previous genome presented for *C. vulgaris* UTEX395 where only 7100 transcripts were predicted (Guarnieri *et al*., 2018). Consistently, more than 20% of the genes identified in *C. vulgaris* 211/11P was not found in the draft genome of *C. vulgaris* UTEX395 by local BLAST (Figure S2).

To further evaluate the transcriptome quality and completeness, Benchmarking Universal Single‐Copy Orthologs (BUSCO) analysis was performed on a benchmark of 303 genes putatively found in all eukaryotes in single copy: this analysis identified complete information for 289 (95.4%) of orthologs and fragmented information for 3 (1%), while only 11 genes (3.6%) were missing, demonstrating a high completeness of the *de novo* assembled genome. Furthermore, when the mRNA‐seq libraries were aligned to the genome assembly, 85.58 ± 0.32% of reads aligned uniquely (mean ± SD, *n* = 6) and an additional 11.81 ± 0.37% aligned to multiple locations, indicating that the genome assembly covered nearly all coding genes.

**Figure 4:**
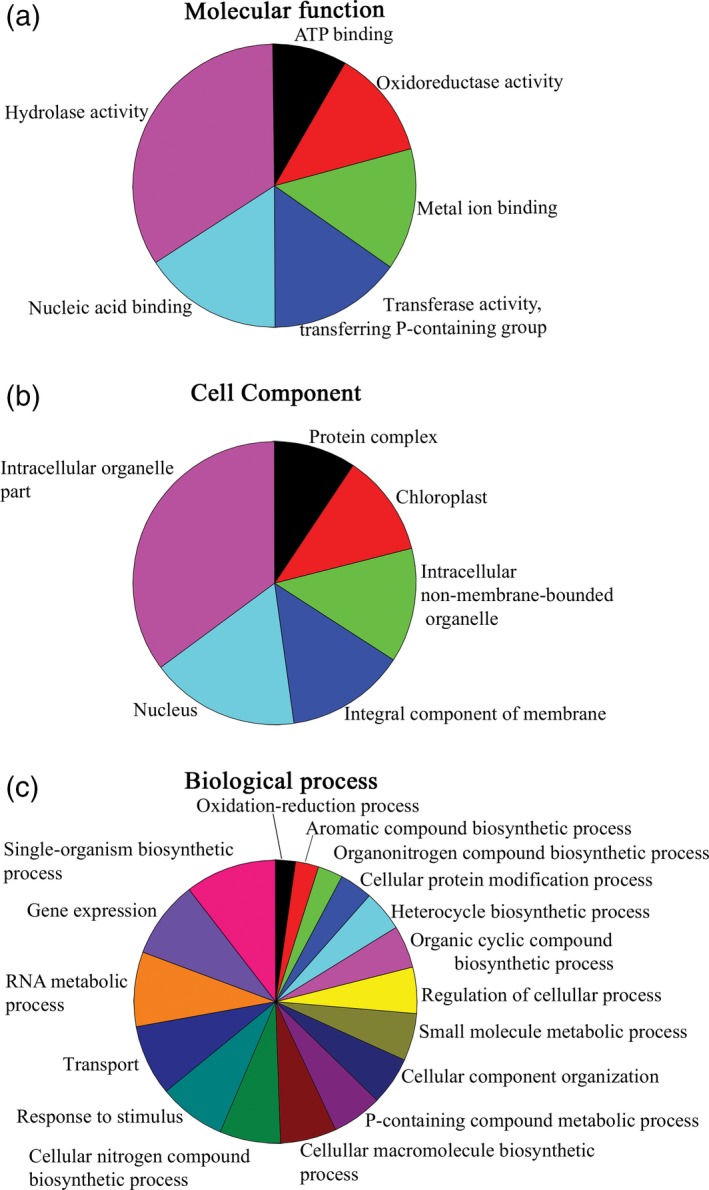
Gene Ontology (GO) classification of annotated *Chlorella vulgaris* 211/11P genes. *Chlorella vulgaris* transcripts annotated using the blast2Go program were functionally grouped based on the GO terms ‘molecular function’ (a), ‘cellular component’ (b) and ‘biological process’ (c). The distribution of the different groups is reported based on the node score associated to each group considering GO term with node score higher than 1%.

Functional genome annotation performed by BLAST2GO analysis reported 5642 associated to Gene Ontology (GO) terms (Figure 4). As reported in Figure S3, considering the top‐hit species distribution, most of the *C. vulgaris* 211/11P genes (~71% of the total genes) were annotated with genes from *Chlorella variabilis*, followed by *Auxenochlorella protothecoides* and *Coccomyxa subellipsoidea*. Functional annotation of the *C. vulgaris* 211/11P genome was then exploited for the analysis of the phylogenies of the 211/11P strain. In particular, 111 single‐copy genes shared with other species with an available genome were used for protein alignment and phylogenetic tree construction. As reported in Figure S4, *C. vulgaris* 211/11P is closely related to *C. vulgaris* UTEX395 strain and to other species from the *Chlorella* genus as *C. variabilis* and *A. protothecoides*.

### Chloroplast and mitochondrial genomes

**Figure 5:**
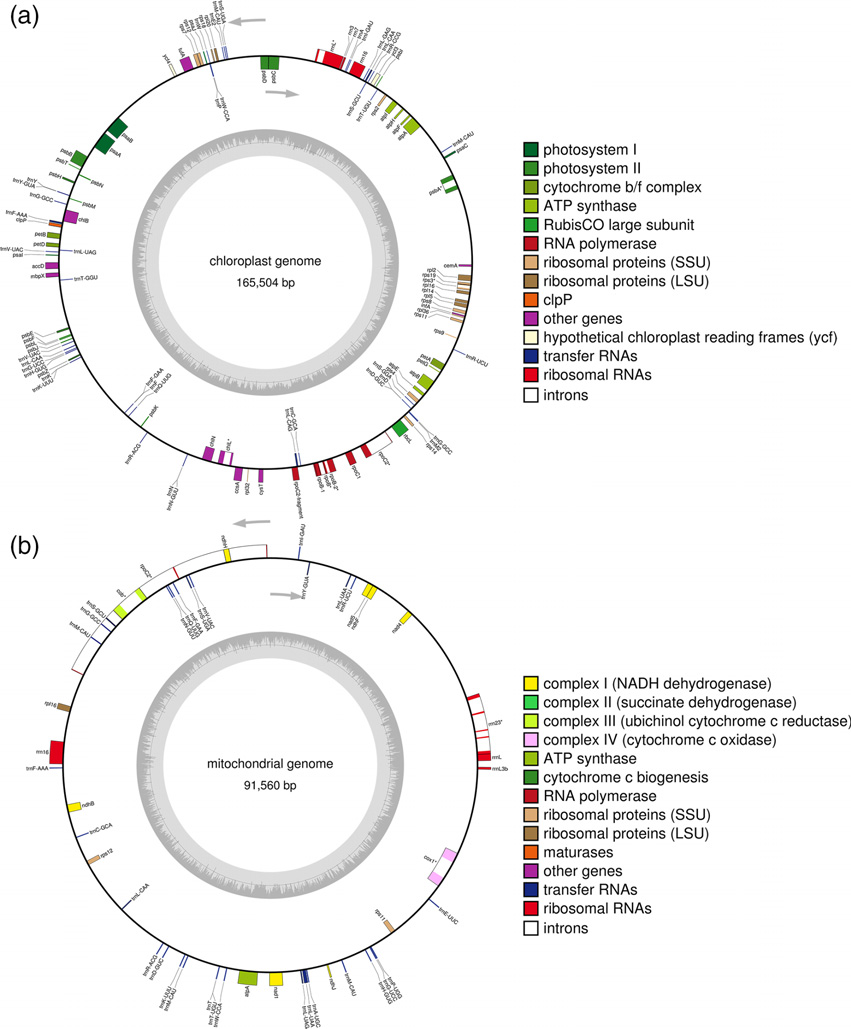
*Chlorella vulgaris* 211/11P chloroplast and mitochondrial genomes. Chloroplast (a) and mitochondrial (b) genomes were assembled based on PacBio data. Location of putative genes and their sense of transcription are indicated.

Complete (circular) chloroplast genome of *C. vulgaris* 211/11P was reconstructed with no gaps or ambiguous nucleotides and is 165 504 bp in length with 121 genes encoded (Figure 5 and Table 2). The overall GC content of the chloroplast genome is 32%, increased to 35% in coding sequence. Among the genes found in the chloroplast genome six genes encode for rRNA, 18 for ribosomal proteins, 46 genes encode for tRNA, seven genes are component of RNA polymerase and two genes encode for a translation initiation factor (*infA*) and a protein elongation factor Tu (*tufA*). In total, 31 genes were identified encoding for subunits of the complexes involved in the light phase of photosynthesis (PSI, PSII, cytochrome b6f and ATP synthase) and a gene for the large subunit of RUBISCO was also identified. Among the other genes present in the *C. vulgaris* 211/11P chloroplast genome, *ycf1−ycf4* were identified with the *ycf3* and *ycf4* involved in PSI assembly (Boudreau *et al*., 1997). Genes involved in plastid division as minD and minE were also found in the chloroplast genome, as previously reported for other *Chlorella* spp. (Wakasugi *et al*., 1997). Notably, three introns were identified in genes *psbA*,* rpoC2* and *rrnL* as previously reported for *C. reinhardtii* (Maul *et al*., 2002). *RpoB*,* chlL and rps3* genes were also found to contain introns.

**Table 2:** Comparison of *Chlorella vulgaris* 211/11P chloroplast and mitochondrial genome with other known microalgae genomes

Chloroplast genome	*Chlorella vulgaris* 211/11P (this work)	*Chromochloris zofingiensis* (Roth *et al*., 2017)	*Chlamydomonas reinhardtii* (v5.5) (Merchant *et al*., 2007; Blaby *et al*., 2014)	*Chlorella variabilis* NC64A (Blanc *et al*., 2010)	*Nannochloropsis gaditana* B‐31 (Corteggiani Carpinelli *et al*., 2014)
Sequenced genome size	165 kbp	181 kbp	204 kbp	125 kbp	115 kbp
Annotated protein‐coding genes	71	71	67 + 1 (tscA)	79	93
Annotated rRNAs	4	6	10	3	6
Annotated tRNAs	46	31	29	31	28
% G+C	31.7%	31%	34%	34%	54.2%

Mitochondrial genome	*Chlorella vulgaris* 211/11P (this work)	*Chromochloris zofingiensis* (Roth *et al*., 2017)	*Chlamydomonas reinhardtii* (v5.5) (Merchant *et al*., 2007; Blaby *et al*., 2014)	*Chlorella variabilis* NC64A (Blanc *et al*., 2010)	*Nannochloropsis gaditana* B‐31 (Corteggiani Carpinelli *et al*., 2014)
Sequenced genome size	92 kbp	42 kbp	16 kbp	78 kbp	42 kbp
Annotated protein‐coding genes	14	22	8	32	5
Annotated rRNAs	4	6	14	3	2
Annotated tRNAs	30	24	3	27	26
% G+C	29.8%	36%	45%	28%	54.2%

*C. vulgaris* 211/11P mitochondrial genome was entirely reconstructed as having a 91 560 bp size with 48 genes encoded, for a total of 16% of the coding region (Figure 5, Table 2). The large size of *C. vulgaris* 211/11P mitochondrial DNA is consistent with the mitochondrial genomes of other green algae as *C. zofingiensis* or higher plants, but significantly larger than the mitochondrial genome of some other green algae such as *Chlamydomonas eugametos* or *C. reinhardtii* (Denovan‐Wright *et al*., 1998; Roth *et al*., 2017). The increased size of *C. vulgaris* 211/11P mitochondrial genome is largely due to the occurrence of high level of non‐coding sequences (84%). Among the genes in the mitochondrial genome, four genes encode for rRNA and 30 for tRNA, while three genes encode for ribosomal proteins. Seven genes encoding for subunits of complex I, and two genes for complexes III and IV subunits (*cob* and *cox1* respectively) were also identified together with a gene for alpha subunit of mitochondrial ATP synthase. Notably, a pseudogene *rpoC2* was found also in the mitochondrial genome, even if with a low expression profile (*rpoC2*). *rpoC2* was usually found in the chloroplast genome coding for a RNA polymerase beta subunit (Shimada *et al*., 1990) but traces of *rpoC2* plastid gene in the mitochondrial genome have been already reported occasionally in mitochondrial genome of land plants (Goremykin *et al*., 2009; Straub *et al*., 2013). This result suggests an uncommon horizontal gene transfer from chloroplast to mitochondrial genome in *Chlorophyta* which was previously reported only upon land colonization (Wang *et al*., 2007; Gandini and Sanchez‐Puerta, 2017).

### Differential gene expression in high light versus low light conditions

RNA‐seq data allowed us to identify the genes which were differently expressed in LL versus HL. In particular, 684 genes were upregulated in LL, while 816 genes were upregulated in HL. GO classification of differentially expressed genes is reported in Figure S5. Putative transcription factors upregulated in LL or HL are reported in Table S6 with 23 genes and 16 genes, respectively, being identified. The genes showing the highest differential transcription (highest log2FC) in LL or in HL are reported in Table S7: genes encoding for chlorophyll binding proteins, nitrate transporter, ferric reductase, protein involved in the cell cycle and a subunit involved in mRNA stability were identified among the genes upregulated in LL. These findings agree with the higher content of chlorophyll per cells in LL, and with the involvement of nitrogen and iron metabolism involved in the biosynthesis of these pigments. In HL the genes showing the strongest upregulation were those encoding proteins for lipoylation, acetyl‐CoA synthetase, early light‐inducible chloroplastic‐like proteins, H(+) hexose cotransporter and a cryptochrome subunits. Genome annotation and analysis of differentially expressed genes were then used to elucidate the different metabolic pathways and their changes in LL versus HL. Some of these pathways are detailed in the following sections.

### Identification of genes involved in key metabolic pathways

The functional annotation of the *C. vulgaris* 211/11P genome allowed the identification of genes coding for the key enzymes involved in the different metabolic pathways of the cell, such as glycolysis, gluconeogenesis, the tricarboxylic acid (TCA) and glyoxylate cycles, photosynthesis, lipid and pigment metabolism (Table S8–S10). Genes involved in some critical metabolic pathways and cellular functions are described in detail here.

#### Photosynthesis

All genes, except for the *psbX* gene, encoding subunits of the membrane complexes or soluble electron carriers involved in the light phase of photosynthesis, are encoded either by nuclear or chloroplast genomes of *C. vulgaris* 211/11P (Table S8). Genes encoding for PSII core subunits were identified in the chloroplast and nuclear genome, in agreement with previous data reported for *A. thaliana* and *C. reinhardtii* (Daniell *et al*., 2016). No homologous gene could be found in the *C. vulgaris* 211/11P genome except for the *psbX* gene. PSBX is a low molecular mass subunit of Photosystem II, which has been reported previously in higher plants and in some algae (Shi *et al*., 1999; Shi and Schröder, 2004). Antisense genotypes on this subunit in *A. thaliana* or knockout mutants in cyanobacteria were characterized by a 30–40% reduction of PSII accumulation, but no apparent growth phenotype was observed, suggesting this subunit is not essential for the photosynthetic process (Shi *et al*., 2012).

For the PSI complex, all core subunits were identified with the exception of PsaM and PsaX: PsaM has been previously reported in cyanobacteria, in some green algae, mosses and gymnosperms but not angiosperms, whereas PsaX has only been found in cyanobacteria (Scheller *et al*., 2001). Different genes were identified in *C. vulgaris* 211/11P genome encoding for Light Harvesting Complexes (LHC), the pigment‐binding antenna proteins bound to the periphery of photosystems devoted to light harvesting and photoprotection.

While both LHCII and LHCI type complexes could be identified, being bound to PSII and PSI respectively, no gene coding for a LHCB6 (CP24) protein was found, supporting that this PSII antenna proteins appeared only in land plant, in agreement with previous finding (Kouřil *et al*., 2016). Interestingly a homolog for the LHCB4 (CP29) subunit could not be found in the *C. vulgaris* UTEX395 genome (Table S8). Most of the genes encoding for LHC complexes were heavily downregulated in HL, in agreement with the reduced chlorophyll content per cell observed in this condition (Figure 1).

Notably both the LHC‐like subunits PSBS and LHCSR were found in *C. vulgaris* 211/11P encoded by single genes: these subunits are involved in the photoprotective mechanism known as non‐photochemical quenching (NPQ), where a significant portion of the excitation energy absorbed by photosystems is thermally dissipated (Li *et al*., 2000; Peers *et al*., 2009). Distinct from *C. reinhardtii*, where LHCSR subunits are strongly overexpressed in HL (Peers *et al*., 2009), *lhcsr* gene in *C. vulgaris* 211/11P is constitutively expressed either in LL‐ or HL‐grown cells (Table S8). Also, for *psbs*,* C. vulgaris* 211/11P behaves differently compared with *C. reinhardtii*: in the latter *psbs* is only transiently expressed under UV or HL conditions (Allorent *et al*., 2016; Correa‐Galvis *et al*., 2016; Tibiletti *et al*., 2016), whereas in *C. vulgaris* 211/11P the *psbs* gene is always expressed but upregulated in HL (Table S8) for *A. thaliana* (Ballottari *et al*., 2007). These results suggested a different regulation of NPQ in *C. vulgaris* 211/11P compared with *C. reinhardtii*, even if the potential role of LHCSR and PSBS in NPQ induction in the former require additional confirmatory experiments.

Genes encoding for protein subunits reported in *C. reinhardtii* and involved in alternative chloroplast electron transport pathways are present in the *C. vulgaris* 211/11P genome, but not differently expressed in LL or in HL (Table S8), such as PGRL1 (Petroutsos *et al*., 2009), PGR5 (Johnson *et al*., 2014) and a type II calcium‐dependent NADH dehydrogenase (NDA2) (Desplats *et al*., 2009; Saroussi *et al*., 2016), involved in cyclic electron flow or PTOX involved in chlororespiration (Rumeau *et al*., 2007; Houille‐Vernes *et al*., 2011).

For the dark phase of photosynthesis and carbon fixation, all subunits previously reported to be involved in this pathway have been identified (Table S8) but generally not differently expressed in LL versus HL. Only phosphoglycerate kinase and ribose 5‐phosphate isomerase showed upregulation in LL: these enzymes are also involved in the oxidative pentose phosphate pathway, which might be more relevant in LL than in HL.

Notably, based on the KEGG Mapper tool, all enzymes required for a C4‐like carbon fixation pathway are present in the *C. vulgaris* 211/11P genome (Figure S6), with the key enzyme involved in carbon fixation in C4 compound phosphoenolpyruvate carboxylase (PPC), encoded by two genes g3928 and g4635, predicted in the cytosol and in the mitochondria, respectively. These two isoforms of PPC might have a role in oxaloacetate formation in the anaplerotic reactions, or for gluoconeogenesis or as alternative carbon fixation to RUBISCO, as previously suggested for *C. sorokiniana* (Cecchin *et al*., 2018).

#### Carotenoid biosynthesis

Carotenoid biosynthetic genes were identified in the *C. vulgaris* 211/11P genome (Table S8). Each of the genes involved in carotene and xanthophyll biosynthesis was found in single copy with some particular genes such as prolycopene isomerase, ζ‐carotene desaturase and ζ‐carotene isomerase being identified in the *C. vulgaris* 211/11P but not in the *C. vulgaris* UTEX395 genome. Notably, a gene coding for neoxanthin synthase could be identified in *C. vulgaris* 211/11P (Figure S7), catalyzing the synthesis of neoxanthin from violaxanthin (Dall'Osto *et al*., 2007), even if further experimental evidence are required to support the enzymatic activity of the putative neoxanthin synthase herein identified. Most of the genes encoding for enzymes involved in carotenoid biosynthesis were present in higher levels in HL (Table S8), in agreement with the increased carotenoid content per cell identified in this condition (Figure 1). In the *C. vulgaris* 211/11P genome no gene coding for a beta‐carotene ketolase (BKT) was identified. This is the key enzyme together with a hydroxylase (CRTZ) for astaxanthin biosynthesis from beta‐carotene or zeaxanthin in different algal species known to accumulate astaxanthin as *Haematococcus lacustris* (formerly known as *Haematococcus pluvialis)* or *C. zofingiensis* (Zhong *et al*., 2011). Whereas CTRZ is present in *C. vulgaris* 211/11P and upregulated in HL (gene g8453), the absence of BKT explains the absence of astaxanthin in this organism and suggests the possibility of biotechnological manipulation to induce the accumulation of this carotenoid that would have increased value to the market for *C. vulgaris*.

#### Glycolysis, gluconeogenesis and oxidative pentose phosphate pathway

Complete set of genes encoding for glycolysis and gluconeogenesis were retrieved in the *Chlorella vulgaris* 211/11P genome. Most of these genes were not differently expressed in LL versus HL except for genes coding a chloroplastic phosphoglycerate kinase and a cytoplasmic fructose‐bisphosphate aldolase, and fructose‐1,6‐bisphosphatase I, which were all downregulated in HL. While fructose‐bisphosphate aldolase might be involved in both glycolysis and gluconeogenesis, fructose‐1,6‐bisphosphatase I is specifically involved in gluconeogenesis, leading to the accumulation of fructose‐6P (Rufty and Huber, 1983). Considering the cytoplasmic predicted localization of both enzymes fructose‐bisphosphate aldolase and fructose‐1,6‐bisphosphatase I upregulated in LL, a possible downregulation of cytosolic gluconeogenesis in HL can be proposed as a consequence of redirection of carbon flow towards lipid accumulation (Figure 1). The upregulation of chloroplastic phosphoglycerate kinase in LL is consistent with upregulation of the gene encoding ribose 5‐phosphate isomerase (Table S8). These enzymes are involved in both carbon fixation and the oxidative pentose phosphate pathway and their upregulation in LL might be related to reduced photosynthetic NADPH formation at low irradiance, therefore with increased requirement for NADPH formation by oxidative pentose phosphate pathway. Further experimental evidence is required to support this hypothesis.

#### TCA cycle and glyoxylate cycle

**Figure 6:**
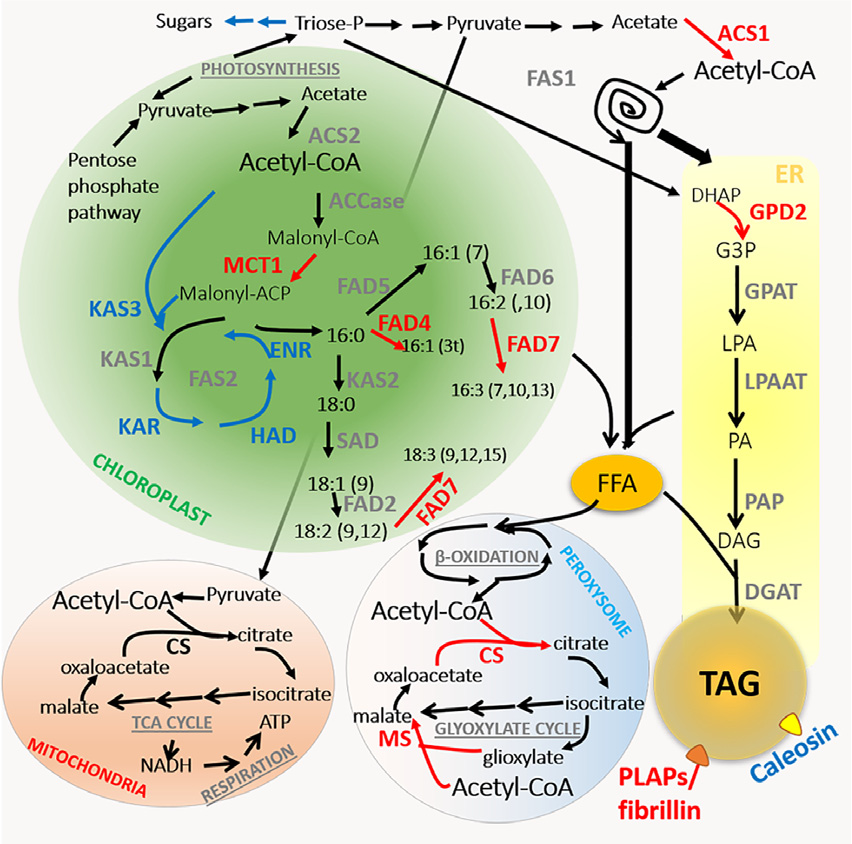
A schematic model of lipid biosynthesis in *Chlorella vulgaris 211/11P*. Proteins encoded by differently expressed genes are indicated in red (upregulated in HL) or in blue (downregulated in HL). Similar color code is used for chemical reactions catalyzed by differently expressed enzymes. ACCase, acetyl‐CoA carboxylase; MCT, malonyl‐CoA: ACP transacylase; KAS, 3‐ketoacyl‐ synthase; KAR, 3‐oxoacy‐ACP reductase; HAD, 3‐hydroxyacyl‐ACP dehydratase; ENR, enoyl‐ACP reductase; FAS1/2, fatty acid synthase type 1/2; SAD, stearate desaturase; FAD2, ω‐6 fatty acid desaturase, Δ12; FAD4, Δ3 palmitate desaturase; FAD5, palmitate Δ7 desaturase; FAD6, ω6 fatty acid desaturase; FAD7, chloroplast glycerolipid ω3 fatty acid desaturase; FFA, free fatty acids; CS, citrate synthase; MS, malate synthase ACS, acetyl‐CoA synthase; DHAP, dihydroxyacetone phosphate; GPD, glycerol‐3‐phosphate dehydrogenase; G3P, glycerol‐3‐phosphate; GPAT, Glycerol‐3‐phosphate *O*‐acyltransferase; LPA, lysophosphatidic acid; LPAAT, lysophosphatidic acid acyltransferase; PA, phosphatidic acid; PAP, phosphatidate phosphatase; DAG, diacylglycerol; DGAT, diacylglycerol acyltransferase; PLAPs/fibrillin, Plastid‐lipid‐associated protein PAP/fibrillin family protein; ER, endoplasmic reticulum; TAG, triacyclglycerols.

Genes encoding for enzymes involved in the TCA cycle and the glyoxylate cycle were identified in *C. vulgaris* 211/11P. Among these genes, differential expression in LL versus HL was observed only for two genes encoding citrate synthase and malate synthase, both upregulated in HL. Interestingly both enzymes catalyze reactions in which acetyl‐CoA is a substrate (Figure 6): citrate synthase catalyzes acetyl‐CoA binding to oxaloacetate, forming citrate as the initial step in the TCA cycle or in the glyoxylate cycle. Malate synthase is involved in the glyoxylate cycle, catalyzing malate formation from acetyl‐CoA and glyoxylate (Boyle and Morgan, 2009; Plancke *et al*., 2014). Glyoxylate cycle has been reported to be in ancestral peroxisomes in *C. reinhardtii* (Kong *et al*., 2017) but its localization should be further investigated in *C. vulgaris*. HL acclimation therefore stimulated upregulation of genes encoding for enzymes involved in acetyl‐CoA consumption, which is likely to be more abundant at high irradiance due to increased carbon fixation and increased sugar production.

#### Lipid biosynthesis

Genes encoding key proteins of lipid metabolism were identified in *C. vulgaris* 211/11P genome, most of which showed alterations in transcription upon HL exposure (Table S9). Consistent with increased TAG accumulation under HL (Figure 1), the genes encoding enzymes involved in earlier steps of *de novo* fatty acid synthesis, the formation of glycerol‐3‐phosphate (G3P) and TAG packaging proteins were upregulated (Table S9). Intriguingly, no change in the expression of genes involved in polar membrane lipid synthesis nor TAG assembly enzymes was observed. Among the highly upregulated genes was a gene coding for acetyl‐CoA synthetase (ACS). ACS is involved in the pyruvate dehydrogenase bypass pathway, by which acetyl‐CoA is produced by glycolytic pyruvate through the intermediates acetaldehyde and acetate (Lin and Oliver, 2008). The importance of ACS enzymes in lipid biosynthesis in plant cells has been demonstrated in *A. thaliana*, in which mutations in *acs* genes caused a strong reduction in plant fitness (Lin and Oliver, 2008). Two genes coding for ACS enzymes were identified in *C. vulgaris* 211/11P, g2176 and g2145, the former being predicted in the cytosol, while the latter in the chloroplast: only the gene encoding the cytosolic ACS was upregulated in HL, suggesting a possible cytoplasmic biosynthesis of fatty acids triggered by HL exposure (Figure 6). However, fatty acid biosynthesis in *Chlorophyta* was generally considered to occur in the chloroplast catalyzed by fatty acid synthase (FAS) type II complex, while cytosolic fatty acid biosynthesis occurs in fungal/animal cells catalyzed by FAS type I complex (Schweizer and Hofmann, 2004). In HL conditions, several genes encoding for FAS type II subunits were downregulated (Figure 6 and Table S9), with the only exception being the chloroplastic malonyl‐CoA: ACP transacylase. Intriguingly, in addition to genes coding for FAS type II subunits, a single large gene encoding for polyketide synthase (PKS)/FAS type I multisubunit complex was also identified (g276). The occurrence of PKS/FAS1‐like complexes in algal cells has already been reported in the oleaginous species *Nannochloropsis oceanica* and *Nannochloropsis gaditana*, and suggested to be involved in cytosolic biosynthesis of fatty acids (Vieler *et al*., 2012; Poliner *et al*., 2015; Alboresi *et al*., 2016). For *Chlorophyta*, several PKS multisubunit complexes have been reported (Poliner *et al*., 2015; Heimerl *et al*., 2018) (Figure S8), even if their function is still under debate. For *C. reinhardtii*, a PKS complex has been reported to be expressed only during zygote formation and to be involved in zygote maturation (Heimerl *et al*., 2018). The putative PKS/FAS type I gene identified in *C. vulgaris* 211/11P is 55 kbp and contains all the t protein domains required for fatty acid biosynthesis containing 11 β‐ketoacyl synthetase domains, nine ketoreductase domains, nine dehydroreductase domains and six enoylreductase domains (Smith and Tsai, 2007) (Schweizer and Hofmann, 2004) (Figure S8). The absence of an acyltransferase domain can be compensated for by the cytoplasmic isoform of malonyl‐CoA: ACP transacylase enzyme (Figure S9), which catalyzes the acyltransferase reaction required for fatty acid biosynthesis by the PKS/FAS type I multisubunit complex identified. Consistent with the increase in several polyunsaturated fatty acids (Figure 2), upregulation under HL of *FAD4* (Zäuner *et al*., 2012) and *FAD7* (Nguyen *et al*., 2013) encoding genes was found.

In addition to *de novo* fatty acid biosynthesis, enzymes involved in the supply of glycerol backbones and TAG packaging were upregulated in HL. Two G3P dehydrogenase (GPD) enzymes were indeed identified in the *C. vulgaris* 211/11P genome, but only the non‐chloroplastic GPD2 was upregulated in HL: this enzyme has been reported in *C. reinhardtii* to be involved in supplying G3P for TAG synthesis and accumulation in nutrient starvation (Driver *et al*., 2017).

HL acclimation led to an increased accumulation of several plastid (PLAP/fibrillin) lipid‐associated proteins: these subunits have been reported to be involved in the formation of lipid droplets (LDs) observed in cells accumulating neutral lipids or carotenoids (Youssef *et al*., 2010), which are both strongly increased under HL in *C. vulgaris* 211/11P (Figure 1). Surprisingly, no genes encoding oleosin or major lipid droplet protein (MLDP) were identified in *C. vulgaris*; these proteins are the main LD‐associated proteins reported in higher plants (Murphy, 2012) and green algae (Moellering and Benning, 2010), respectively. Two genes were identified encoding for caleosin, a calcium‐binding protein that can be found in multicellular plants and green algae, and frequently described as an LD‐associated protein (Lin *et al*., 2012; Charuchinda *et al*., 2015). Among the two caleosin genes identified in *C. vulgaris* 211/11P, none was upregulated in HL, but rather the g8244 gene was downregulated under these conditions. This result suggested that LD formation under HL involved not only *de novo* synthesis of fatty acids and TAG accumulation but also synchronization with LD packaging proteins.

### Identification of genes involved in meiosis and motility

*C. vulgaris* strains have been usually described as non‐motile and asexual (Yamamoto *et al*., 2004). Genes previously reported to be associated with meiosis and motility were searched for in the *C. vulgaris* 211/11P genome. Previously DMC1 and Rad51 DNA recombinase homologs were reported in the *C. vulgaris* genome (Guarnieri *et al*., 2018). In addition to DMC1 and Rad51 DNA recombinase homologs, the main genes involved in meiosis (Malik *et al*., 2007) were present and transcribed in the *C. vulgaris* 211/11P genome (Table S8) and this situation is similar to that previously reported for other green algae such as *C. zofingiensis* (Roth *et al*., 2017) or *C. variabilis* NC64A (Blanc *et al*., 2010) in which sexual reproduction is cryptic and not well defined. This result suggested a possible sexual reproductive stage also in *C. vulgaris* 211/11P with gamete formation. In agreement with this finding, a gene in the *C. vulgaris 211/11P* genome encoding gametolysin was found (g3347), together with a gene encoding a protein containing a domain with a putative CGS1/HAP2 function and that is essential for cell fusion (Blanc *et al*., 2010; Wong and Johnson, 2010) (Figure S10). The genes involved in motility were then investigated by comparison with the genes listed in CiliaCut, a group of genes identified in *C. reinhardtii* involved in the formation of sensory or motility cilia and flagella (Merchant *et al*., 2007). Among the 195 genes in CiliaCut, 114 genes were identified in *C. vulgaris* 211/11P (58.4%). In particular, 78.2% of the genes in CiliaCut that were present in the diatom *Thalassiosira pseudonana* were present also in *C. vulgaris* 211/11P (Table S10): 84.2% of the *T. pseudonana* genes in MotileCut (genes in CiliaCut involved in motile flagella functions) were present also in *C. vulgaris* 211/11P. This result suggested that *C. vulgaris* 211/11P might be able to form gametes with motile flagella as previously observed for *T. pseudonana* during gametogenesis (Moore *et al*., 2017).

## Discussion

Integration of highly accurate Illumina sequencing with long‐reads (PacBio) and optical mapping (Bionano Genomics) allowed us to obtain the assembled genome of *C. vulgaris* 211/11P in 14 scaffolds with a relatively good N50 of 2.8 Mb. This is a >100‐fold improvement compared with the recently released *C. vulgaris* genome (Guarnieri *et al*., 2018) (Table S4). We can speculate that the 14 pseudo‐molecules reconstructed may represent the chromosomes of *C. vulgaris* 211/11P, with 98.9% of scaffolded genome, a much higher percentage compared with most other available genomes of green algae (Table 1). The *C. vulgaris* 211/11P genome size of 40 Mbp was close to that of other members of the *Chlorella* genus or closely related species (Table 2). The GC content of the *C. vulgaris* 211/11P genome was similar compared with *C. variabilis* or *C. reinhardtii*, but higher compared with *C. zofingiensis*. The integration of RNA‐seq data allowed us to obtain a detailed functional annotation of the assembled *C. vulgaris* 211/11P genome with a number of transcripts and proteins consistent with the data reported for *C. variabilis*, but almost halved compared with *C. reinhardtii* or *C. zofingesis*, revealing a strong variability in the green lineage. For comparison, for the microalga *Nannochloropsis gaditana* (Heterokonta) with a much smaller genome (23 Mbp) a similar protein number compared with *C. vulgaris* 211/11P was observed. Notably, exon and intron average length and the number of exons per transcript were similar when compared with another member of the *Chlorella* genus, *Chlorella varibilis NC64A*, but shorter compared with *C. reinhardtii* or *C. zofingiensis* (Table 1). It is worth noting that the improved quality of genome and functional annotation of *C. vulgaris* 221/11P strain allowed us to identify 2285 genes that were not previously predicted in *C. vulgaris* UTEX395 strain (Guarnieri *et al*., 2018), among which were genes highly differently expressed in LL versus HL conditions, transcription factors putatively involved in HL or LL acclimation, enzymes involved in carotenoid biosynthesis and genes involved in motility (Tables S6–S10). The absence of these and other genes in the previously reported genome of *C. vulgaris* UTEX395 strain could be related to the fact that *C. vulgaris* 211/11P and UTEX395 strains are different (Figure S5) or to the low quality of the reported *C. vulgaris* UTEX395 genome (Table S4).

The results obtained by genome assembly and functional annotation revealed the presence of some peculiar features in *C. vulgaris* 211/11P in common with higher plants, but different from the model organism for green algae, *C. reinhardtii*. Evidence for horizontal transfer from the chloroplast to the mitochondria could be found in *C. vulgaris* 211/11P, as for fragments of plastid *rpoC2* gene found as a pseudogene in the mitochondrial genome. Chloroplast gene or gene fragments were indeed previously observed only in mitochondria of higher plants, attributed to the earlier event of plastid to mitochondria horizontal gene transfer to the common ancestor of extant angiosperms and gymnosperms: the analysis of *C. vulgaris* 211/11P genome demonstrated that this horizontal gene transfer can be found also in some *Chlorophyta*, but not in the model organism for green algae *C. reinhardtii* (Wang *et al*., 2007). The possible functions of plastid genes in mitochondrial genome is still not clear, being usually not expressed (Wang *et al*., 2007). For *C. vulgaris* 211/11P, the plastid gene *rpoC2* was found in the mitochondrial genome as a fragment with a low expression profile: further experiments are required to investigate its possible role in mitochondrial gene expression.

Another plant‐like feature found in *C. vulgaris* 211/P is the mitochondrial genome size (91.5 kbp), which is larger than the mitochondrial genome of *C. reinhardtii* (15.7 kbp) and more similar to the mitochondrial genome found for higher plants (Blaby *et al*., 2014).

A mixed situation compared with higher plants and other green algae was found in *C. vulgaris* 211/11P considering the genes involved in photoprotection. LHCSR subunits have been reported to be critical for tuning photosynthetic efficiency and photoprotection in microalgae (Peers *et al*., 2009; Berteotti *et al*., 2016) while PSBS has a similar function in higher plants (Li *et al*., 2000). In *C. reinhardtii LHCSR* subunits are upregulated in HL (Peers *et al*., 2009) while *PSBS* is only transiently expressed in HL or upon UV exposure (Allorent *et al*., 2016; Correa‐Galvis *et al*., 2016; Tibiletti *et al*., 2016). In *C. vulgaris* 211/11P both *LHCSR* and *PSBS* genes are expressed in LL and HL, with only PSBS being upregulated in HL (Table S8), adding further evidence of the strongly debated role of PSBS protein also in green algae and not only in higher plants. LHCSR and PSBS accumulation and functioning should be further investigated in detail in *C. vulgaris* to determine their role in photoprotection and HL acclimation.

In other cases, genes found in higher plants and in *C. reinhardtii* were not identified in *C. vulgaris*, as for oleosins: these proteins are the major lipid droplet‐associated proteins found in higher plants, but their conservation was reported also for some green algae as *C. reinhardtii* and *Volvox carterii* (Huang *et al*., 2013). *C. vulgaris* 211/11P was also characterized by the absence of genes encoding MLDP proteins, the major proteins involved in LD formation in green algae (Moellering and Benning, 2010) as for the other green alga species *Auxenochlorella protothecoides* (Lin *et al*., 2012). Rather, caleosins and PLAP/fibrillin proteins were the main proteins involved in LD formation in *C. vulgaris* 211/11P with a strong upregulation of the latter under HL conditions in which TAGs are mainly accumulated (Figure 1). These finding suggested a divergent evolution among green algae, leading to specialized molecular mechanisms at the base of the phenotypes observed with an evolutive pressure driven by interaction with the environment. Caleosins have been reported to be present both in higher plants and in green algae with a peroxidase activity associated that was proposed to be involved in oxylipin production (Charuchinda *et al*., 2015): oxylipins are molecules produced by enzymatic or non‐enzymatic fatty acid oxidation that trigger the cell response to oxidative stress. In LL‐adapted *C. vulgaris 211/11P* cells, caleosin genes were upregulated (Table S9), suggesting their main role in LD formation in LL with the possibility to produce oxylipin upon oxidative stress. In contrast, when cells were acclimated to HL conditions, these gene were downregulated, this was likely to be due to the previous activation of cell mechanisms allowing HL acclimation and prevention of oxidative stress. Further experiments are required to validate the role of caleosins and PLAP/fibrillin proteins in TAGs accumulation and HL acclimation.

HL acclimation led to a strong increase in TAG and a decrease in the main lipids found in thylakoid membranes, such as galactolipids MGDG, DGDG and the phospholipid PG. Reduction of thylakoid lipids in HL is indeed consistent with a decrease in the chlorophyll content per cell and downregulation of the main chlorophyll binding proteins, the LHC (Table S8) in these conditions, as a consequence of increased irradiation and the reduced need for light harvesting. The observed TAG accumulation suggested that increased carbon fixation redirects carbon flow towards fatty acid biosynthesis with strong increase in the HL of palmitic acid (16:0), oleic acid (18:1), linoleic acid (18:2) and α‐linolenic acid (18:3) (Figure 2). These fatty acids are then assembled into TAGs and stored in LDs.

The genomic and transcriptomic data described here allowed us to draw a model based on this phenotype, as discussed (Figure 6), taking into consideration that further events in translation or posttranslational levels could also take place, affecting the highlighted metabolic pathways. Potential dual sites for fatty acid biosynthesis in *C. vulgaris* 211/11P can be proposed based on identification of cytosolic ACS involved in the pyruvate dehydrogenase bypass pathway (Lin and Oliver, 2008), a PKS/FAS type I and a cytosolic malonyl‐CoA: ACP transacylase. Cytosolic lipid production by PKS/FAS type I is a common metabolic pathway in animal and fungal cells but it was recently suggested to be present also in some microalgae species (Vieler *et al*., 2012; Alboresi *et al*., 2016); further experimental evidence is required to support this finding in *C. vulgaris*. The similar starch content per cell observed in HL further suggested that the increased carbon fixation caused increased triose‐P to be used to produce acetyl‐CoA in the plastid and, in the cytosol, to produce precursors for fatty acid biosynthesis. Accordingly, enzymes involved in cytosolic gluconeogenesis were downregulated in HL, suggesting a preferential use of chloroplast‐derived triose‐P to produce pyruvate and acetyl‐CoA. Increased lipid accumulation in *C. vulgaris* 211/11P in HL can therefore be related to increased acetyl‐CoA production in the chloroplast and in the cytosol by ACSs leading to upregulation of enzymes involved in TAG assembly, as the GDP2 enzyme and LD stabilization, as PLAP/fibrillin subunits (Figure 6). Further experiments are required to validate the metabolic model proposed in Figure 6 that, at the present stage, is based only on genomic and transcriptomic data. The different enzymes involved in lipid accumulation (Table S9) and the transcription factor identified as differently expressed in HL versus LL (Table S6) could be potential targets for biotechnological manipulation of *C. vulgaris* to increase lipid production and biomass productivity.

Interestingly, genes involved in sexual reproduction and motility were also identified in *C. vulgaris* 211/11P (Table S10) even if further research activity is required to induce gamete formation and mating under laboratory conditions. Understanding sexual reproduction in this species would be critical for accumulating in the same strain genetic traits for increased productivity.

In conclusion, the assembly and functional annotation of the *C. vulgaris* 211/11P genome potentially enabled the application of genome‐editing technologies in this species, and allowed the identification of potential targets for biotechnological manipulation of this organism, for its exploitation for biomass and high value products or for transferring specific *C. vulgaris* 211/11P properties to other species.

## Experimental procedures

### *Chlorella vulgaris* cultivation

The *C. vulgaris 211/11P* strain was obtained from the Culture Collection of Algae at Goettingen University (CCAP211/11P strain). Cells were grown photoautotrophically in BG‐11 medium at 25°C in flasks in low (70 μmol m^−2^ sec^−1^) or high (1000 μmol m^−2^ sec^−1^) white light irradiation with a 16 h light : 8 h dark photoperiod (Allen and Stanier, 1968).

### Lipid, protein and starch analysis

Due to its strong cell wall, *C. vulgaris 211/11P* cells were first sonicated three times in a solution containing 1 ml EDTA 1 mm and acetic acid 0.15 m. Total lipid was extracted from sonicated cells following the method of Bligh and Dyer (1959). Lipid extracts were separated by thin layer chromatography and quantified for neutral or polar lipids based on densitometry and comparison with known amounts of lipid standards (Siaut *et al*., 2011). For fatty acid composition analysis, one given fraction of the lipid extracts was converted to their fatty acid methyl esters (FAMEs) and then analyzed using gas chromatography–flame ionization detection (GC‐FID) as detailed in Siaut *et al*. (2011). Proteins and starch content of the harvested biomass were analyzed as reported previously in Cecchin *et al*. (2018).

### DNA extraction and quality control

DNA was extracted from 500 ml *C. vulgaris 211/11P* liquid cultures with a cell density of 5 × 10^7^ cell per ml using the cetyltrimethyl ammonium bromide (CTAB) extraction buffer. Extracted DNA was treated with 200 μg ml^−1^ RNase A at 37°C for 20 min and subsequently purified using 1.8× Agencourt AMPure XP beads (Beckman Coulter s.r.l., Milan, Italy). DNA purity and integrity were assessed using a NanoDrop 1000 spectrophotometer (Thermo Scientific, Wilmington, DE, USA) and by capillary electrophoresis on a 2200 TapeStation (Agilent Technologies, Santa Clara, CA, USA), respectively. DNA quantification was performed using the Qubit dsDNA HS Assay kit (Life Technologies, Monza, Italy).

### Illumina sequencing

DNA (500 ng) was fragmented through sonication using a Covaris S220 instrument (Covaris, Woburn, MA, USA) and DNA‐seq libraries were generated using the TruSeq DNA kit according to the manufacturer's instructions (Illumina, San Diego, CA, USA). Library length was assessed by capillary electrophoresis on a 2200 TapeStation (Agilent Technologies) and quantified by qPCR using primers annealing to the adapter sequences. DNA‐seq libraries were sequenced on an Illumina HiSeq1000 platform and generating 100‐bp paired‐end reads for a total of 2.5 Gb.

### PacBio sequencing

Genomic DNA (16 μg) was used for the preparation of two independent single‐molecule real‐time (SMRT) bell libraries according to the manufacturer's protocol (Pacific Biosciences; 20‐kb template preparation using BluePippin, SageScience, Beverly, MA, USA, size selection system with a 15‐kb cut‐off). Sequencing was performed on a PacBio RS‐II platform (Pacific Biosciences, CA, USA) generating 6.4 Gb of SMRT data using PacBio P6‐C4 chemistry.

### BioNano genome mapping

High‐molecular‐weight DNA was extracted from the pellet of 2 L of cell culture with optical density (OD)_750_ = 5.3, corresponding approximately to a total of 3 g. The cell wall was destroyed by grinding in liquid nitrogen. Ground cells were resuspended in IrysPrep Plant Homogenization Buffer (Bionano Genomics) supplemented with 0.2% beta‐mercaptoethanol and 1 mm spermine‐spermidine (HB+) and filtered through a 40‐μm cell strainer. Nuclei were collected by centrifugation at 4500 ***g*** for 20 min at 4°C. A centrifugation at 60 ***g*** for 2 min at 4°C was used to remove debris, whereas nuclei were collected from the supernatant (3500 ***g*** for 20 min at 4°C). Nuclei were further purified by centrifugation over an IrysPrep Density Gradient (Bionano Genomics, San Diego, CA, USA) at 4500 ***g*** for 40 min at 4°C. The nuclei band (white layer) was collected from the gradient interphase and washed two times in HB+ and collected by centrifugation at 2500 ***g*** for 20 min. Only the nuclei pellet (white band) was collected with a wide bore tip and transferred for washing after each centrifugation step. Nuclei were embedded in agarose plugs and high‐molecular‐weight DNA was extracted as previously described (Staňková *et al*., 2016). The mega‐base size of extracted DNA was verified by pulsed‐field electrophoresis (PFGE). DNA (300 ng) was labeled and stained using the Nt.BspQI nicking endonuclease in combination with the –NLRS DNA labeling kit (Bionano Genomics). The nicked and labeled DNA was then loaded onto an IrysChip for imaging on the Irys system (BioNano Genomics) for three runs of 30 cycles in one flow cell. Molecules of <150 kb in length, label SNR <2.75, label intensity >0.6 and having less than 20 labels were removed. Bionano data were assembled into consensus genome maps using the BioNano Solve pipeline (v5678.6119rel) with RefAligner (v.6119).

### Genome assembly

The *C. vulgaris 211/11P* genome was assembled using FALCON (Chin *et al*., 2016) v1.8.7. A second assembly run was performed using the 12% of PacBio subreads that did not align on the first assembly, applying more relaxed parameters. The two assemblies were merged.

PacBio subreads were aligned to the assembly using pbalign (v0.2.0.138342) and then the GenomicConsensus package (v0.9.2) with the Quiver algorithm was used to remove errors present in the consensus sequences. To further improve the genome quality, a second polishing iteration was performed using Illumina data: reads were aligned using BWA‐MEM software (0.7.15‐r1140) and the Pilon (v1.22) tool was used to correct errors.

A hybrid assembly combining the polished PacBio assembly with the optical map was performed with the Bionano Solve Pipeline (v5678.6119rel), RefAligner (v.6119) using a merging‐step *P*‐value of 1e‐11 and a ‘Min alignment length and Max endoutlier’ parameter of 80.

### Organelle genome assembly

The organelle genomes were assembled using the Organelle_PBA pipeline (Soorni *et al*., 2017). The sequences were then polished following the same approach used for the nuclear genome. The alignment between the FALCON assembly and the organelle genomes was performed using Blastn (v2.6.0). Those PacBio contigs aligning to the organelle genome with a similarity of at least of 99% were manually removed.

### RNA extraction and RNA‐seq analysis

RNA was extracted from 500 ml of *C. vulgaris 211/11P* in liquid cultures with a cell density of 7 × 10^7^ cell per ml. RNA quality and quantity were determined using a NanoDrop 2000 spectrophotometer (Thermo Scientific) and a Bioanalyzer Chip RNA 7500 series II (Agilent, Santa Clara, CA, USA), respectively. Directional RNA‐seq library preparation was performed starting from 1 μg total RNA using the TruSeq RNA Sample Prep Kit v2 (Illumina Inc.) after capturing poly‐adenylated transcripts. Library quality was assessed using a High Sensitivity DNA Kit on a 2200 Tape Station (Agilent, Wokingham, UK) and quantification of libraries was performed by qPCR using primers annealing to the adapter sequences. Libraries were sequenced with an Illumina NextSeq500 sequencer (Illumina Inc.) generating ~22 million 75‐bp paired‐end reads per sample.

### Gene annotation

Gene annotation of the nuclear genome was performed using the unsupervised RNA‐seq‐based BRAKER1 pipeline, which takes advantage of two gene predictors: GeneMark‐ET 4.32 and AUGUSTUS 3.0.3 (Specht *et al*., 2011). Briefly, both RNA‐seq data from the two different growth conditions, LL and HL, were used for the annotation. Quality of reads obtained from each sample was assessed using FastQC software (http://www.bioinformatics.babraham.ac.uk/projects/fastqc/) and reads with more than 10% of undetermined bases or more than 50 bases with a quality score <7 were discarded. Reads were then clipped from the adapter sequences using Scythe software version 0.980 (https://github.com/vsbuffalo/scythe), and low‐quality ends (*Q* score <20 on a 10‐nt window) were trimmed with Sickle version 0.940 (https://github.com/vsbuffalo/sickle). The two RNA‐seq data were merged and alignment of reads to the assembled genome was performed using HISAT2 (https://ccb.jhu.edu/software/hisat2/index.shtml) v2.0.1. Finally, the aligned RNA‐seq reads were used as input for the BRAKER1 pipeline. The quality and completeness of the transcriptome were evaluated using BUSCO, http://busco.ezlab.org/) (Simão *et al*., 2015).

The web application GeSeq was used to annotate the organelle genomes with default parameters plus the tRNAscan‐SE activated and selecting *C. reinhardtii* in the NCBI RefSeq database (Tillich *et al*., 2017). Some genes were also manually curated based on RNA‐seq mapped reads. Organelle genome maps were then generated using the OGDRAW tool (Lohse *et al*., 2013).

### Differential expression analysis

RNA‐seq data were filtered as described in the previous section and aligned to the assembled reference genome with HISAT2 (v2.0.1) (Kim *et al*., 2015). Differential expression analysis between the two growth conditions was conducted with DESeq2 (v1.16.1) (Love *et al*., 2014) using the gene annotations generated.

### Transcriptome functional annotation

Transcriptome functional annotation was performed by the Blast2Go platform based on NCBI's RefSeq database (Conesa *et al*., 2005). Annotated sequences were analyzed using the KAAS (KEGG Automatic Annotation Server) platform to obtain KO annotation (Kanehisa and Goto, 2000; Kanehisa *et al*., 2016, 2017). Transcripts differently expressed with KO annotations were visualized using the KEGG Mapper platform, while the remaining transcripts functionally annotated were manually inspected by retrieving the function of the closest homolog gene.

### Phylogenetic analysis

Phylogenetic analysis was performed by BUSCO analysis as previously reported (Waterhouse *et al*., 2017). In particular, 111 single‐copy genes shared with other species with an available genome were used for protein alignment and phylogenetic tree construction. BUSCO 3.0.2 software with the eukaryota_odb9 database and the genome of each species *Chlorella vulgaris*,* Chlorella protothecoides sp0710*,* Chlorella variabilis NC64A*,* Coccomyxa subellipsoidae*,* Chlamydomonas reinhardtii*,* Volvox carteri*,* Chromochloris zofingiensis*,* Arabidopsis thaliana*,* Micromonas pusilla CCMP1545* and *Ostreococcus tauri* were used to identify the single‐copy orthologous genes. Of these, only those shared between the 10 species were selected. For each protein a multiple alignment was performed among the species using MUSCLE 3.8.31 (Edgar, 2004), and then the alignments were concatenated. The tree was built using the web application Phylogeny.fr running PhyMl and TreeDyn for construction and visualization, respectively (Dereeper *et al*., 2008).

### Subcellular localization prediction

Subcellular localization prediction was performed using the PredAlgo tool, as previously described (Tardif *et al*., 2012).

## Accession numbers

This Whole Genome Shotgun project was deposited in the DDBJ/ENA/GenBank database under the accession number SIDB00000000. The version described in this paper is version SIDB01000000. Accession numbers for the raw PacBio and Illumina reads are SRR8083355−SRR8083370.

## Conflicts of Interest

The authors declare no conflicts of interest.

## Author Contributions

MB conceived the study, designed and supervised the experiments. MD designed, coordinated and supervised the *C. vulgaris* genome assembly and annotation. MC prepared all the samples herein analyzed and performed or contributed to all the experiments herein reported. LM and MR performed genome assembly, genome annotation and performed transcriptome and differential gene expression analysis. EC and MR contributed to sample preparation for next generation sequencing and optical mapping. LG contributed to growth experiments and biomass analysis. YL coordinated lipid analysis and contributed to experiment design. SC performed lipid analysis. MB, MD, MR and YL wrote the manuscript. All the authors discussed the results, contributed to data interpretation and commented on the manuscript.

## Supporting information


**Figure S1**. Example of optical mapping‐based scaffolding of *Chlorella vulgaris* 211/11P genome.
**Figure S2**. Number of transcripts identified in *Chlorella vulgaris* 211/11P with BLAST results using *Chlorella vulgaris* UTEX 395 as reference genome.
**Figure S3**. Distribution of *Chlorella vulgaris* 211/11P gene annotation results.
**Figure S4**. Phylogenetic analysis of *Chlorella vulgaris* 211/11P strain.
**Figure S5**. Gene Ontology (GO) classification of *Chlorella vulgaris* 211/11P differently expressed genes in LL versus HL conditions.
**Figure S6**. Carbon fixation pathway in *Chlorella vulgaris* 211/11P identified by KEGG Mapper.
**Figure S7**. Identification of a neoxanthin synthase enzyme in *Chlorella vulgaris* 211/11P.
**Figure S8**. Polyketide synthase/fatty acid type I enzyme in *C. vulgaris* 211/11P.
**Figure S9**. Protein alignment of two malonyl‐CoA:ACP transacylase enzymes identified in *C. vulgaris* 211/11P.
**Figure S10**. Alignment of *Chlorella vulgaris* 211/11P g3658 gene product with HAP2 from *Chamydomonas reinhardtii*.

**Table S1**. Summary of raw PacBio and Illumina sequencing data and Bionano mapping data.
**Table S2**. *Chlorella vulgaris* 211/11P genome assembly statistics.
**Table S3**. Single‐nucleotide variants (SNV) and insertion−deletion (InDel) in the *Chlorella vulgaris* 211/11P assembled genome before and after correction with Illumina and PacBio data.
**Table S4**. Comparison of *Chlorella vulgaris* genomes reported for strain UTEX 395 and 211/11P.
**Table S5**. Codon usage in *Chlorella vulgaris* 211/11P.

**Table S6**. Identification of *Chlorella vulgaris* 211/11P transcription factor differently expressed in low light (LL) versus high light (HL) conditions.
**Table S7**. Identification of *Chlorella vulgaris* 211/11P most differently expressed genes in low light versus high light.
**Table S8**. Identification of key genes involved in different metabolic pathway in *Chlorella vulgaris* 211/11P.
**Table S9**. Identification of key genes involved in lipid biosynthesis and degradation in *Chlorella vulgaris* 211/11P.
**Table S10**. Identification of genes involved in flagella and cilia formation in *Chlorella vulgaris* 211/11P according to the CiliaCut list.
