# Supplementary material for: Chlorella vulgaris genome assembly and annotation reveals the molecular basis for metabolic acclimation to high light conditions
Source: Plant J. 2019 Sep 24;100(6):1289–305. doi: 10.1111/tpj.14508 (PMC6972661; doi:10.1111/tpj.14508)
Supplement: Supplementary file 1 — Figure S1. Example of optical mapping‐based scaffolding of Chlorella vulgaris 211/11P genome. Figure S2. Number of transcripts identified in Chlorella vulgaris 211/11P with BLAST results using Chlorella vulgaris UTEX 395 as reference genome. Figure S3. Distribution of Chlorella vulgaris 211/11P gene annotation results. Figure S4. Phylogenetic analysis of Chlorella vulgaris 211/11P strain. Figure S5. Gene Ontology (GO) classification of Chlorella vulgaris 211/11P differently expressed genes in LL versus HL conditions. Figure S6. Carbon fixation pathway in Chlorella vulgaris 211/11P identified by KEGG Mapper. Figure S7. Identification of a neoxanthin synthase enzyme in Chlorella vulgaris 211/11P. Figure S8. Polyketide synthase/fatty acid type I enzyme in C. vulgaris 211/11P. Figure S9. Protein alignment of two malonyl‐CoA:ACP transacylase enzymes identified in C. vulgaris 211/11P. Figure S10. Alignment of Chlorella vulgaris 211/11P g3658 gene product with HAP2 from Chamydomonas reinhardtii. [file TPJ-100-1289-s001.docx]

**Figure S1. Example of optical mapping-based scaffolding of *Chlorella vulgaris* 211/11Pgenome.** PacBio contigs and Bionano consensus map are colored in blue, while hybrid assembly in green; vertical lines represent the recognition sites of the enzyme Nt.BspQI used for the insertion of the fluorescent probes in the isolated DNA molecules for the generation of the optical maps.

**
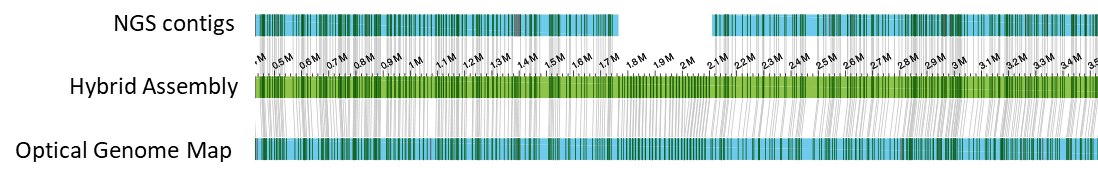
**

**Figure S2. Number of transcripts identified in *Chlorella vulgaris* 211/11P based on BLAST results using *Chlorella vulgaris* UTEX 395 as a reference genome.** Selected threshold value for e-value was set to 1x10^-3^.

**Figure S3. Distribution of *Chlorella vulgaris* 211/11P gene annotation results.** The top-Hit species distribution obtained by functional annotation of *C. vulgaris* genome by BLAST2GO software is reported.

**
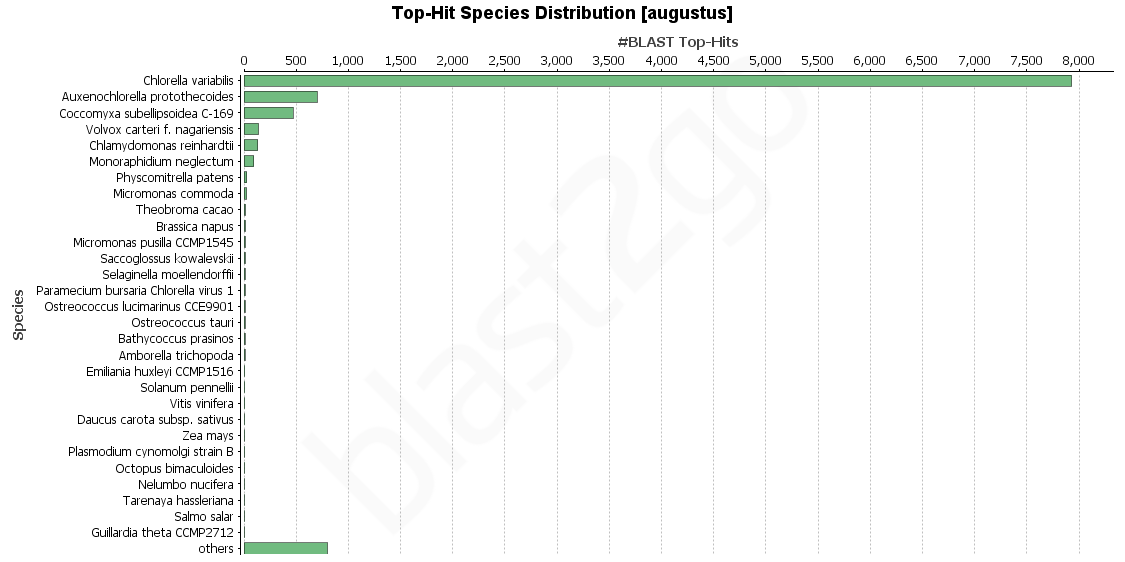
**

**Figure S4. Phylogenetic analysis of *Chlorella vulgaris* 211/11P strain.**


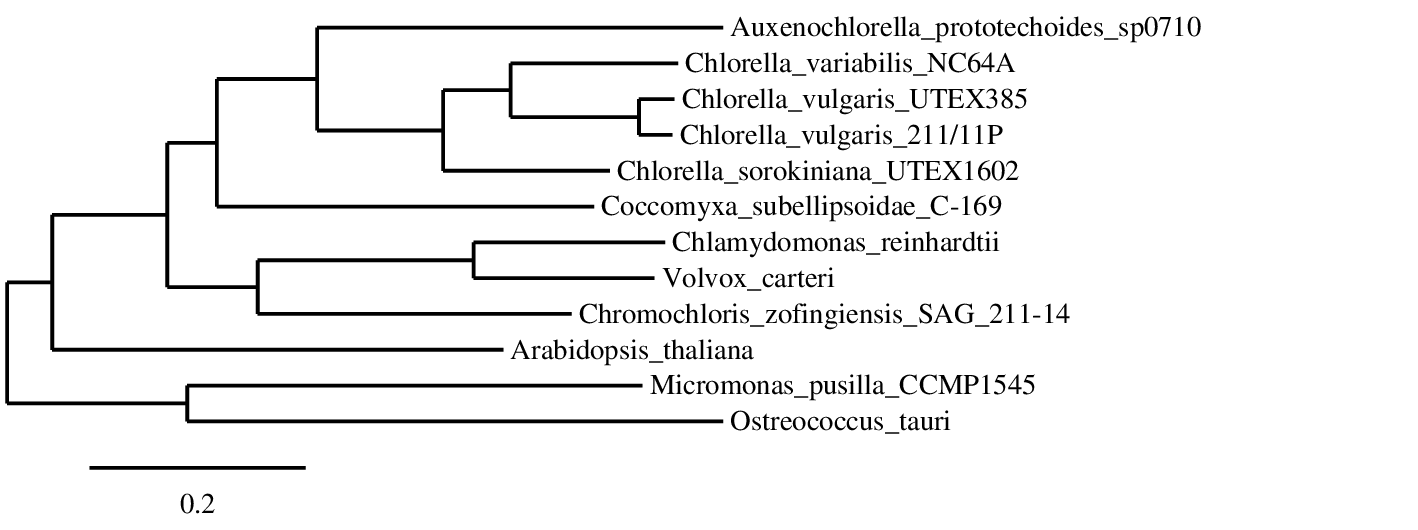


**Figure S5. Gene Ontology (GO) classification of *Chlorella vulgaris* 211/11P** **differently expressed genes in LL vs. HL conditions.** Differentially expressed genes up-regulated in low light (LL) (a, b, c) or high light (HL) (d, e, f) were functionally grouped on the basis of GO terms cellular component (a, d), molecular function (b, e) and biological processes (c, f). The distribution of the different groups is reported based on the node score associated to each group considering GO term with node score higher than 1%.

**
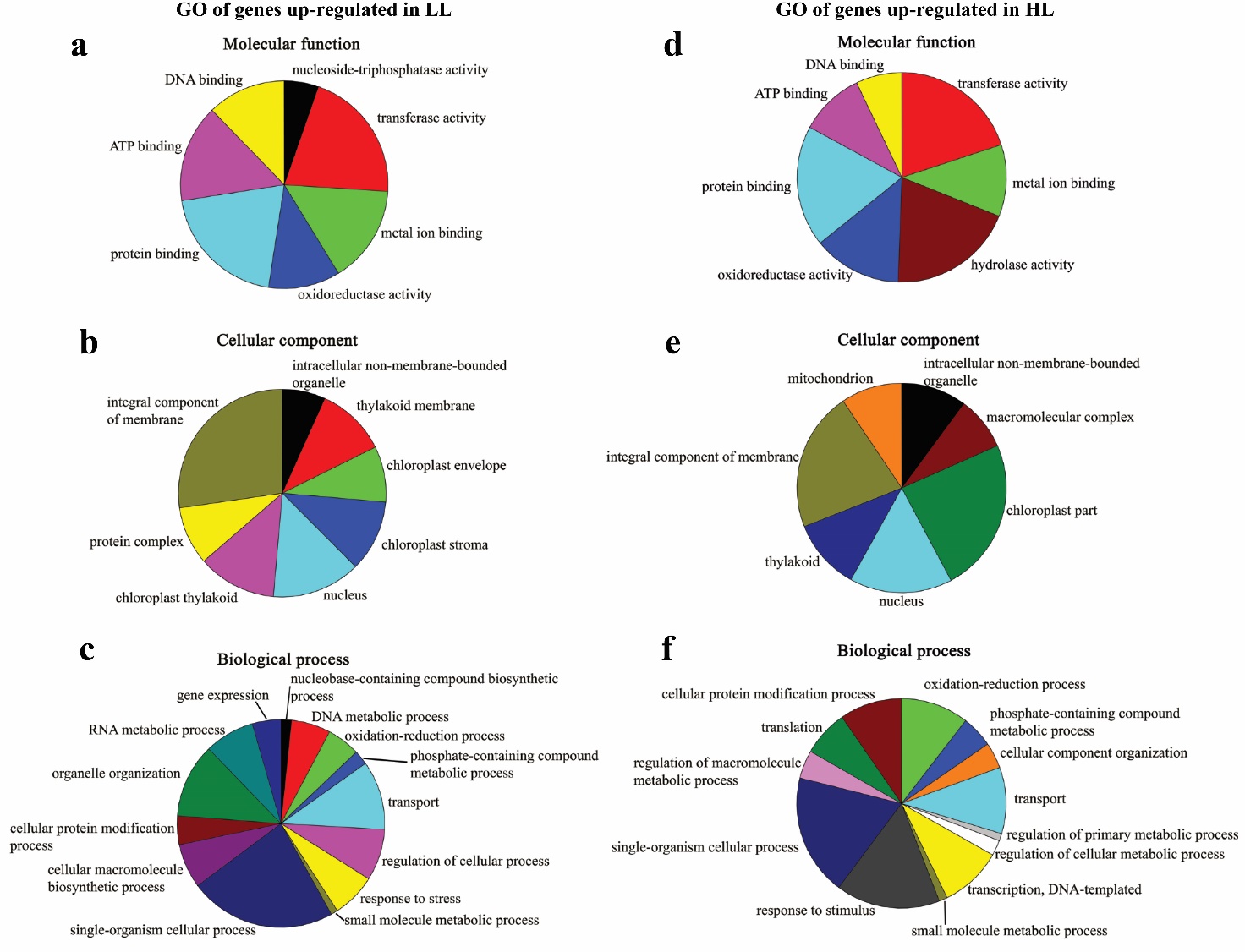
**

**Figure S6. Carbon fixation pathway in *Chlorella vulgaris* 211/11P identified by KEGG Mapper.** Carbon fixation map by KEGG Mapper (map00710) is reported. The enzymes identified in *C. vulgaris* genome are reported in green.


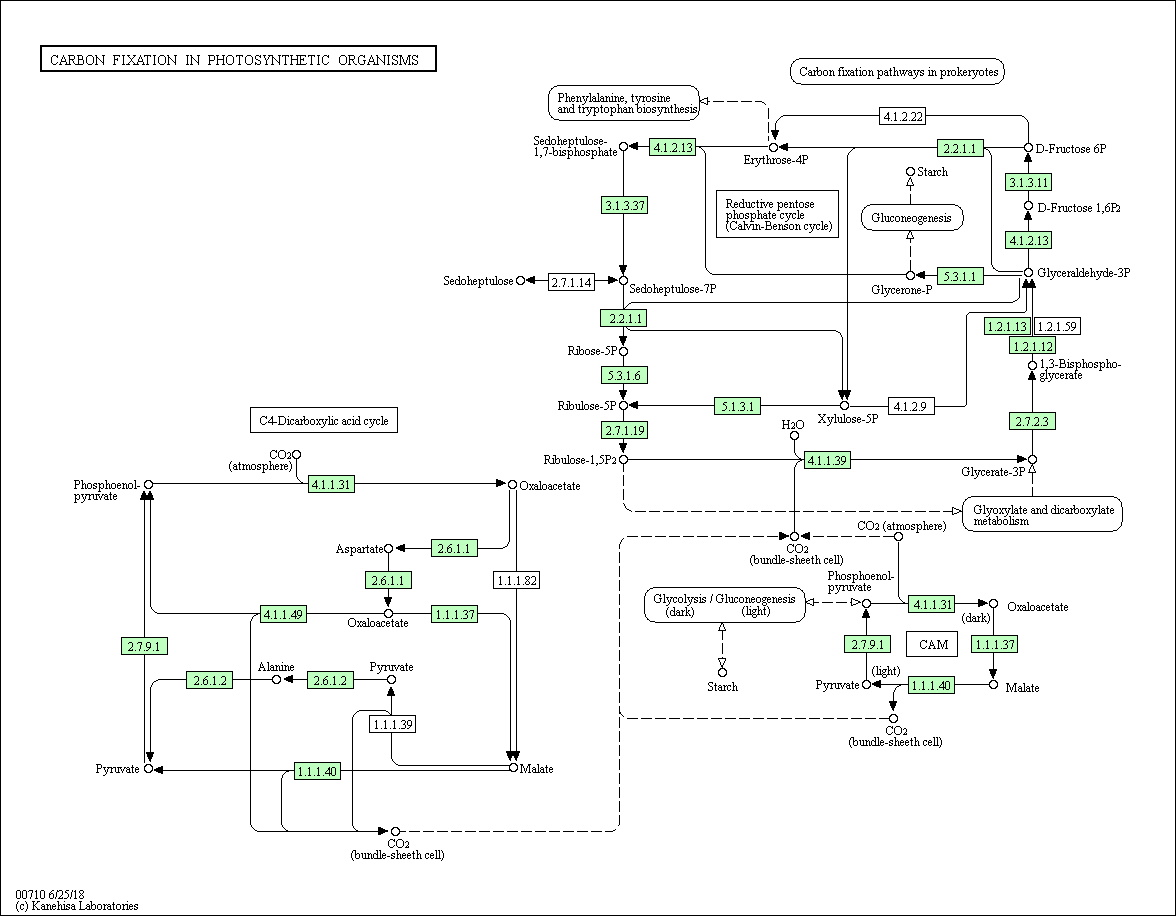


**Figure S7. Identification of a neoxanthin synthase enzyme in *Chlorella vulgaris* 211/11P*.*** Panel A: Clustal Omega protein alignment of the protein sequence encoded by *Chlorella vulgaris* 211/11P gene g5367 and the protein sequence identified in *Arabidopsis thaliana* as neoxanthin synthase. Panel B: representation of the domain identified in the putative neoxanthin synthase of *C. vulgaris* and the identified neoxanthin synthase encoded in the *A. thaliana* genome.

**
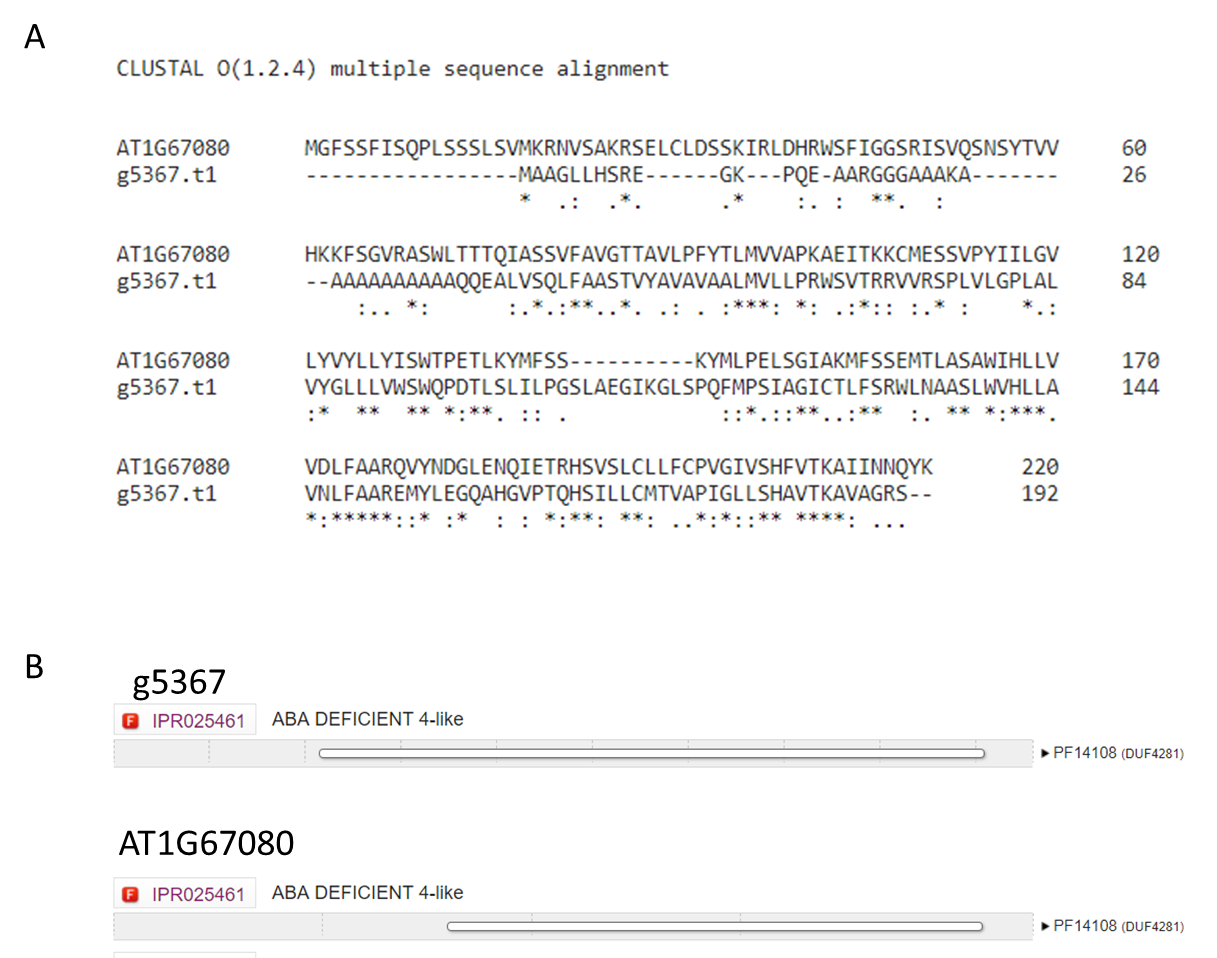
**

**Figure S8. Polyketide synthase/fatty acid synthase Type I enzyme in *C. vulgaris* 211/11P**. Panel A: phylogenetic tree of putative PKS/FAS type I enzyme found in *C. vulgaris* 211/11P (g276.t1), highlighted in yellow). Panel B: protein domains identified by INTERPROSCAN in g276 gene product.

**
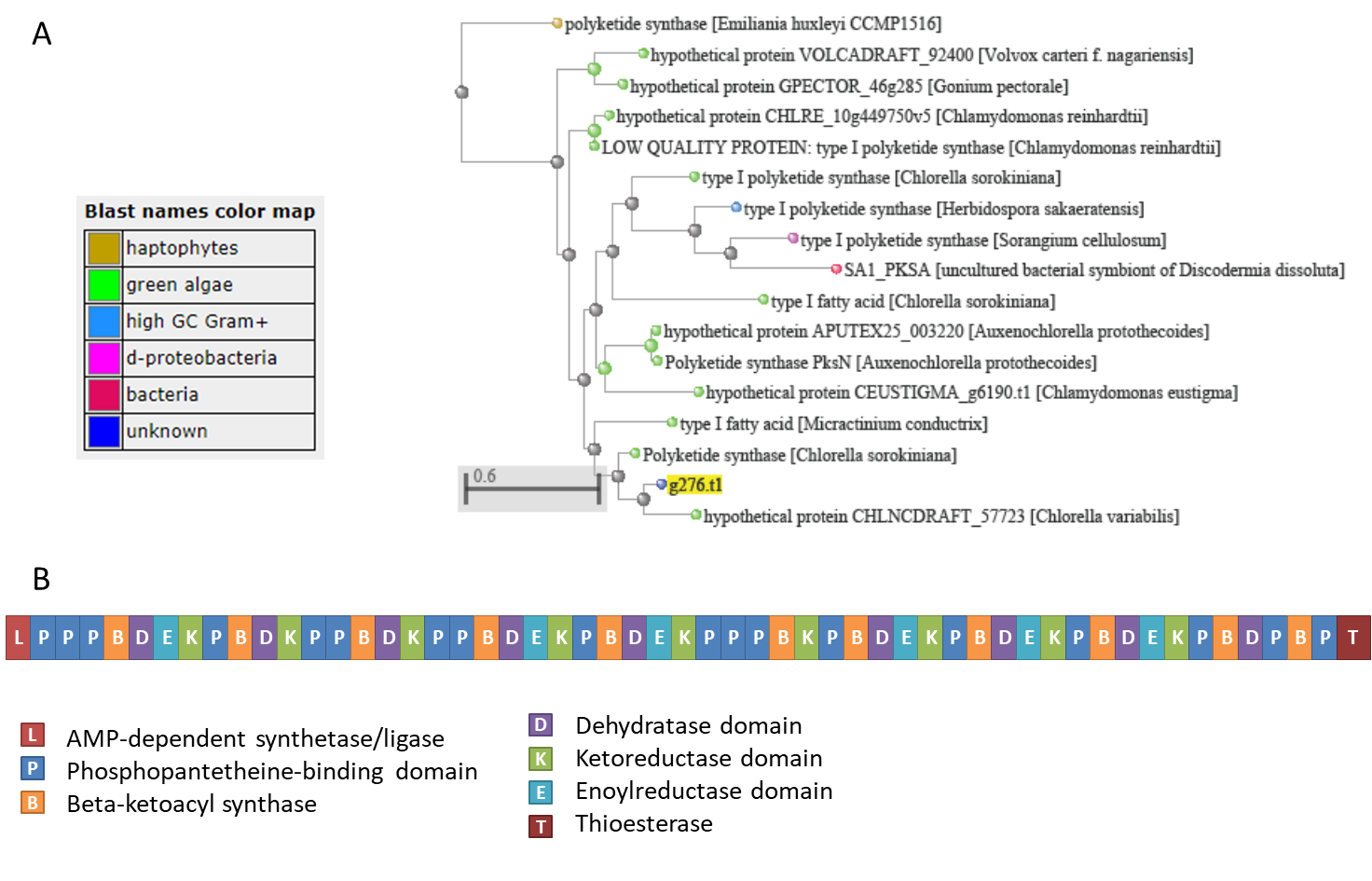
**

**Figure S9. Protein alignment of two Malonyl-CoA: ACP transacylase enzymes identified in *C. vulgaris* 211/11P.** In the case of g6284.t1 transit peptide for chloroplast import is indicated as predicted by PREDALGO software.

**
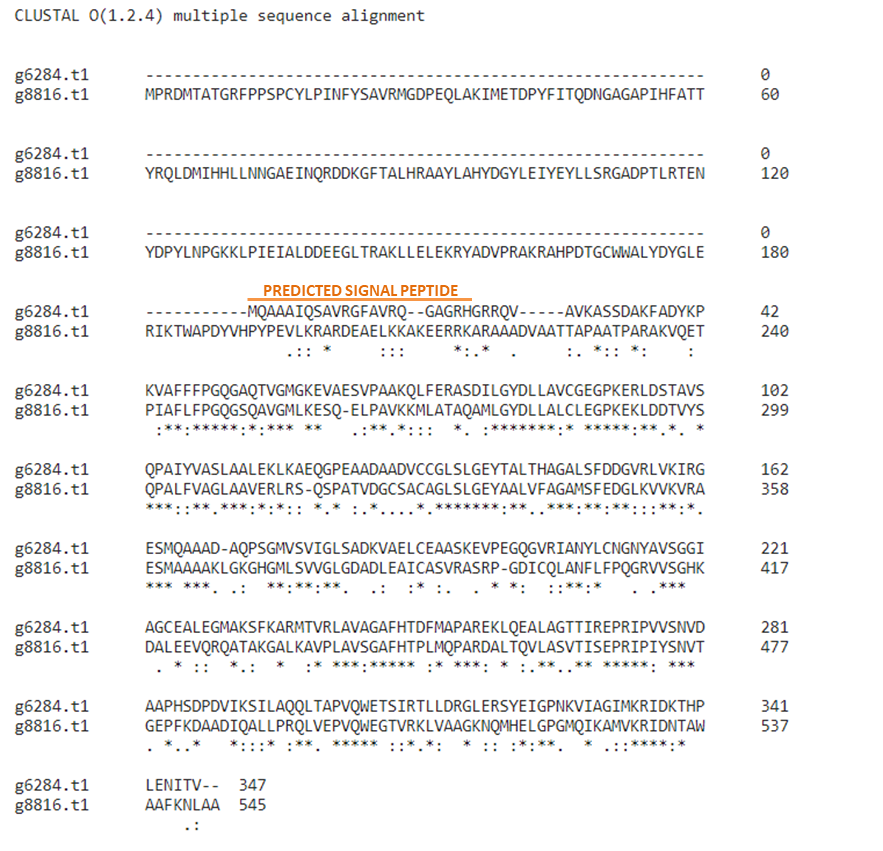
**

**Figure S10. Alignment of *Chlorella vulgaris* 211/11P g3658 gene product with HAP2 from *Chlamydomonas reinhardtii.*
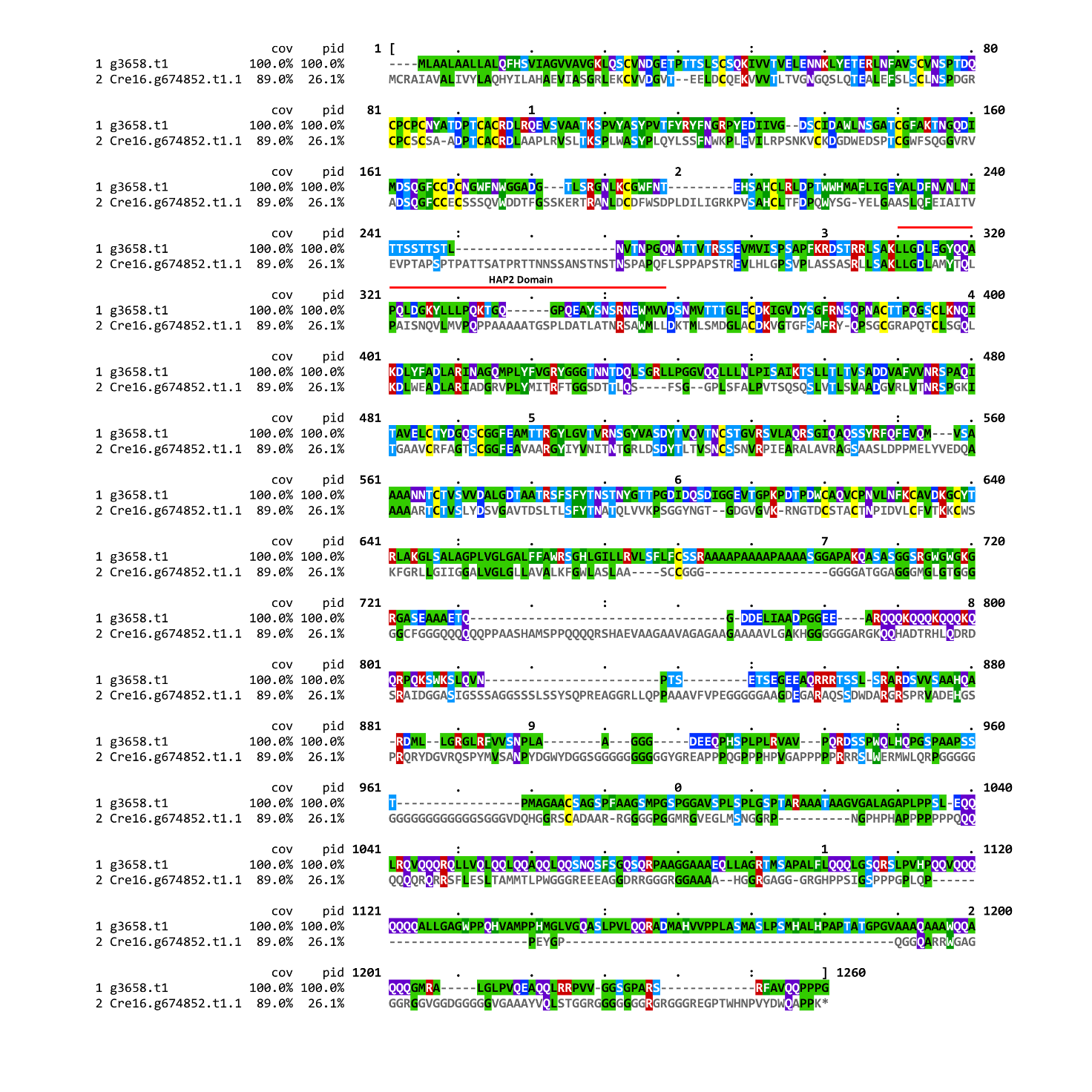
**
