# Supplementary material for: Chlorella vulgaris genome assembly and annotation reveals the molecular basis for metabolic acclimation to high light conditions
Source: Plant J. 2019 Sep 24;100(6):1289–305. doi: 10.1111/tpj.14508 (PMC6972661; doi:10.1111/tpj.14508)
Supplement: Supplementary file 2 — Table S1. Summary of raw PacBio and Illumina sequencing data and Bionano mapping data. Table S2. Chlorella vulgaris 211/11P genome assembly statistics. Table S3. Single‐nucleotide variants (SNV) and insertion−deletion (InDel) in the Chlorella vulgaris 211/11P assembled genome before and after correction with Illumina and PacBio data. Table S4. Comparison of Chlorella vulgaris genomes reported for strain UTEX 395 and 211/11P. Table S5. Codon usage in Chlorella vulgaris 211/11P. [file TPJ-100-1289-s002.docx]

**Table S1:** **Summary of raw PacBio and Illumina sequencing data and Bionano mapping data.**

|  | **PacBio** | **Illumina** | **Bionano** |
| --- | --- | --- | --- |
| **Number of bases (Gb)** | 6.4 | 2.5 | 69.0 |
| **Number of reads** | 1,113,721 | 25,428,036 | 566,536 |
| **Genome coverage** | 128X | 50X | 1380X |
| **Mean read length (bp)** | 5,784 | 100 | 121,810 |
| **N50 (in bp)** | 8,757 | 100 | 191,600 |

**Table S2. *Chlorella vulgaris* 211/11P genome assembly statistics.** *Two round assembly merged, not polished

|  | **Falcon assembly*** | **Bionano consensus genome Map** | **Hybrid assembly** | **Unplaced** | **Chloroplast** | **Mitochondrion** | **Hybrid assembly + Unplaced without organelle contamination** |
| --- | --- | --- | --- | --- | --- | --- | --- |
| **Total assembly length (bp)** | 39,785,701 | 171,505 | 39,740,717 | 440,075 | 165,504 | 91,583 | 40,180,792 |
| **Number of sequences** | 62 | 255 | 14 | 29 | 1 | 1 | 43 |
| **Sequence average length (bp)** | 641,704 | 673,000 | 2,838,622 | 15,175 | 165,504 | 91,583 | 934,437 |
| **Sequence N50 (bp)** | 1,800,706 | 1,049,000 | 2,825,136 | 29,425 | 165,504 | 91,583 | 2,825,136 |
| **Sequence L50** | 8 | 54 | 6 | 4 | 1 | 1 | 6 |
| **Sequence N90 (bp)** | 795,186 | 346,446 | 2,150,204 | 6,908 | 165,504 | 91,583 | 2,150,204 |
| **Sequence L90** | 19 | 160 | 12 | 16 | 1 | 1 | 12 |
| **Largest sequence (bp)** | 5,417,522 | 5,015,440 | 5,422,624 | 128,459 | 165,504 | 91,583 | 5,422,624 |
| **Smallest sequence (bp)** | 416 | 50,112 | 795,975 | 416 | 165,504 | 91,583 | 416 |
| **% GC content** | 61.6 | // | 60.2 | 59.4 | 31.7 | 29.8 | 60.0 |
| **Number of gap** | 0 | // | 12 | 0 | 0 | 0 | 12 |
| **Total gap length (bp)** | 0 | // | 634,943 | 0 | 0 | 0 | 634,943 |

**Table S3. Single nucleotide variants (SNV) and insertion-deletion (InDel) in the *Chlorella vulgaris* 211/11P assembled genome before and after correction with Illumina and PacBio data.**

|  | **PacBio** | **PacBio+Illumina** |
| --- | --- | --- |
| **SNV** | 3076 | 81 |
| **InDel** | 32821 | 190 |
| **TOTAL** | 35897 | 271 |
| **% GENOME** | 0.09% | 0.0007% |

**Table S4. Comparison of *Chlorella vulgaris* genomes reported for strain UTEX 395 and 211/11P.**

|  | *Chlorella vulgaris* UTEX 395 (Guarnieri et al 2018) | *Chlorella vulgaris*  211/11P (this work) |
| --- | --- | --- |
| Total sequence length | 37,342,230 | 40,437,856 |
| Total assembly gap length | 40,625 | 634,943 |
| Gaps between scaffolds | 0 | 0 |
| Number of scaffolds | 3,600 | 14 |
| Scaffold N50 | 27,824 | 2,825,136 |
| Scaffold L50 | 358 | 6 |
| Number of contigs | 4,754 | 45 |
| Contig N50 | 20,333 | 1,802,178 |
| Contig L50 | 501 | 8 |
| Number of component sequences (WGS or clone) | 3,600 | 43 |

**Table S5. Codon usage in *Chlorella vulgaris* 211/11P**. The codon usage table gives for each codon: i. Sequence of the codon. ii. The encoded amino acid. iii. The proportion of usage of the codon among its redundant set, i.e. the set of codons which code for this codon's amino acid. iv. The expected number of codons, given the input sequence(s), per 1000 bases. v. The observed number of codons in the input sequences.

#Codon AA Fraction Frequency Number

GCA A 0.253 37.206 219120

GCC A 0.299 44.031 259312

GCG A 0.257 37.744 222285

GCT A 0.191 28.126 165643

TGC C 0.807 13.076 77007

TGT C 0.193 3.132 18448

GAC D 0.673 30.175 177711

GAT D 0.327 14.646 86253

GAA E 0.174 9.821 57841

GAG E 0.826 46.536 274064

TTC F 0.574 15.009 88392

TTT F 0.426 11.137 65592

GGA G 0.101 8.659 50998

GGC G 0.579 49.753 293009

GGG G 0.194 16.684 98255

GGT G 0.126 10.870 64018

CAC H 0.757 17.008 100166

CAT H 0.243 5.454 32123

ATA I 0.120 3.013 17743

ATC I 0.610 15.291 90055

ATT I 0.270 6.762 39822

AAA K 0.144 4.609 27143

AAG K 0.856 27.343 161029

CTA L 0.030 3.019 17777

CTC L 0.149 14.949 88040

CTG L 0.636 63.856 376067

CTT L 0.072 7.198 42392

TTA L 0.008 0.852 5017

TTG L 0.104 10.452 61556

ATG M 1.000 19.628 115598

AAC N 0.784 16.095 94787

AAT N 0.216 4.424 26056

CCA P 0.210 13.228 77906

CCC P 0.322 20.337 119770

CCG P 0.265 16.701 98356

CCT P 0.204 12.849 75669

CAA Q 0.149 9.126 53746

CAG Q 0.851 51.994 306208

AGA R 0.036 2.264 13336

AGG R 0.133 8.447 49746

CGA R 0.096 6.071 35754

CGC R 0.383 24.222 142651

CGG R 0.260 16.441 96828

CGT R 0.092 5.836 34368

AGC S 0.431 31.268 184148

AGT S 0.068 4.947 29137

TCA S 0.105 7.604 44785

TCC S 0.169 12.228 72014

TCG S 0.127 9.212 54252

TCT S 0.101 7.292 42943

ACA T 0.220 10.108 59529

ACC T 0.392 18.005 106035

ACG T 0.232 10.661 62786

ACT T 0.156 7.150 42107

GTA V 0.052 3.318 19543

GTC V 0.201 12.871 75804

GTG V 0.635 40.676 239551

GTT V 0.112 7.183 42301

TGG W 1.000 13.320 78445

TAC Y 0.754 13.708 80730

TAT Y 0.246 4.481 26391

TAA * 0.086 0.164 964

TAG * 0.210 0.398 2346

TGA * 0.703 1.331 7840
