# Supplementary material for: Chlorella vulgaris genome assembly and annotation reveals the molecular basis for metabolic acclimation to high light conditions
Source: Plant J. 2019 Sep 24;100(6):1289–305. doi: 10.1111/tpj.14508 (PMC6972661; doi:10.1111/tpj.14508)
Supplement: Supplementary file 4 [file TPJ-100-1289-s004.docx]

**Table S1:** **Summary of raw PacBio and Illumina sequencing data and Bionano mapping data.**

**Table S2. *Chlorella vulgaris* 211/11P genome assembly statistics.** *Two round assembly merged, not polished

**Table S3. Single nucleotide variants (SNV) and insertion-deletion (InDel) in the *Chlorella vulgaris* 211/11P assembled genome before and after correction with Illumina and PacBio data.**

**Table S4. Comparison of *Chlorella vulgaris* genomes reported for strain UTEX 395 and 211/11P.**

**Table S5. Codon usage in *Chlorella vulgaris* 211/11P**. The codon usage table gives for each codon: i. Sequence of the codon. ii. The encoded amino acid. iii. The proportion of usage of the codon among its redundant set, i.e. the set of codons which code for this codon's amino acid. iv. The expected number of codons, given the input sequence(s), per 1000 bases. v. The observed number of codons in the input sequences.

**Table S6. Identification of *Chlorella vulgaris* 211/11P transcription factor differently expressed in low light (LL) vs. high light (HL) conditions**

**Table S7. Identification of *Chlorella vulgaris* 211/11P most differently expressed genes in low light vs. high light**

**Table S8. Identification of key genes involved in different metabolic pathway in *Chlorella vulgaris* 211/11P.**

**Table S9. Identification of key genes involved in lipid biosynthesis and degradation in *Chlorella vulgaris* 211/11P.**

**Table S10. Identification of genes involved in flagella and cilia formation in *Chlorella vulgaris* 211/11P according to the CiliaCut list.**

**Figure S1. Example of optical mapping-based scaffolding of *Chlorella vulgaris* 211/11Pgenome.** PacBio contigs and Bionano consensus map are colored in blue, while hybrid assembly in green; vertical lines represent the recognition sites of the enzyme Nt.BspQI used for the insertion of the fluorescent probes in the isolated DNA molecules for the generation of the optical maps.

**Figure S2. Number of transcripts identified in *Chlorella vulgaris* 211/11P based on BLAST results using *Chlorella vulgaris* UTEX 395 as a reference genome.** Selected threshold value for e-value was set to 1x10^-3^.

**Figure S3. Distribution of *Chlorella vulgaris* 211/11P gene annotation results.** The top-Hit species distribution obtained by functional annotation of *C. vulgaris* genome by BLAST2GO software is reported.

**Figure S4. Phylogenetic analysis of *Chlorella vulgaris* 211/11P strain.**

**Figure S5. Gene Ontology (GO) classification of *Chlorella vulgaris* 211/11P** **differently expressed genes in LL vs. HL conditions.** Differentially expressed genes up-regulated in low light (LL) (a, b, c) or high light (HL) (d, e, f) were functionally grouped on the basis of GO terms cellular component (a, d), molecular function (b, e) and biological processes (c, f). The distribution of the different groups is reported based on the node score associated to each group considering GO term with node score higher than 1%.

**Figure S6. Carbon fixation pathway in *Chlorella vulgaris* 211/11P identified by KEGG Mapper.** Carbon fixation map by KEGG Mapper (map00710) is reported. The enzymes identified in *C. vulgaris* genome are reported in green.

**Figure S7. Identification of a neoxanthin synthase enzyme in *Chlorella vulgaris* 211/11P*.*** Panel A: Clustal Omega protein alignment of the protein sequence encoded by *Chlorella vulgaris* 211/11P gene g5367 and the protein sequence identified in *Arabidopsis thaliana* as neoxanthin synthase. Panel B: representation of the domain identified in the putative neoxanthin synthase of *C. vulgaris* and the identified neoxanthin synthase encoded in the *A. thaliana* genome.

**Figure S8. Polyketide synthase/fatty acid synthase Type I enzyme in *C. vulgaris* 211/11P**. Panel A: phylogenetic tree of putative PKS/FAS type I enzyme found in *C. vulgaris* 211/11P (g276.t1), highlighted in yellow). Panel B: protein domains identified by INTERPROSCAN in g276 gene product.

**Figure S9. Protein alignment of two Malonyl-CoA: ACP transacylase enzymes identified in *C. vulgaris* 211/11P.** In the case of g6284.t1 transit peptide for chloroplast import is indicated as predicted by PREDALGO software.

**Figure S10. Alignment of *Chlorella vulgaris* 211/11P g3658 gene product with HAP2 from *Chlamydomonas reinhardtii.***
